# Bacterial Microbiota of Rice Roots: 16S-Based Taxonomic Profiling of Endophytic and Rhizospheric Diversity, Endophytes Isolation and Simplified Endophytic Community

**DOI:** 10.3390/microorganisms6010014

**Published:** 2018-02-11

**Authors:** Felix Moronta-Barrios, Fabrizia Gionechetti, Alberto Pallavicini, Edgloris Marys, Vittorio Venturi

**Affiliations:** 1 Bacteriology Group, International Center for Genetic Engineering and Biotechnology ICGEB, 34149 Trieste, Italy; felix.moronta@icgeb.org; 2 Laboratory of Plant Biotechnology and Virology, Center for Microbiology and Cell Biology, Venezuelan Institute of Scientific Research IVIC, Caracas 1020A, Venezuela; edgloris@gmail.com; 3 Department of Life Science, University of Trieste, 34127 Trieste, Italy; fgionechetti@units.it (F.G.); pallavic@univ.trieste.it (A.P.)

**Keywords:** rice, endophyte, sustainable agriculture, plant microbiome, simplified bacterial community, syncom, taxonomic profiling, core plant microbiome

## Abstract

Rice is currently the most important food crop in the world and we are only just beginning to study the bacterial associated microbiome. It is of importance to perform screenings of the core rice microbiota and also to develop new plant-microbe models and simplified communities for increasing our understanding about the formation and function of its microbiome. In order to begin to address this aspect, we have performed a 16S rDNA taxonomic bacterial profiling of the rhizosphere and endorhizosphere of two high-yield rice cultivars—Pionero 2010 FL and DANAC SD20A—extensively grown in Venezuela in 2014. Fifteen putative bacterial endophytes were then isolated from surface-sterilized roots and further studied in vitro and *in planta*. We have then performed inoculation of rice seedlings with a simplified community composed by 10 of the isolates and we have tracked them in the course of 30 days in greenhouse cultivation. The results obtained suggest that a set was able to significantly colonize together the rice endorhizospheres, indicating possible cooperation and the ability to form a stable multispecies community. This approach can be useful in the development of microbial solutions for a more sustainable rice production.

## 1. Introduction

Rice is the staple food for more than a half of the world population and its production is dependent on chemical fertilizers and pesticides [1] which are in part responsible for global warming and groundwater pollution [2]. To meet the world’s demand for rice it is imperative to find environmentally sound ways that supplement the need for fertilizers [3]. The use of microbial inoculants is attractive because they can complement and mitigate the use of the agrochemicals ensuring a healthier environment [2]. 

Microorganisms play an important role in agricultural systems where they live in close association with plants and can exert different kinds of positive effects on its health and growth [4]. Rhizosphere bacteria, which live in the soil that is in intimate contact with the roots, are able to perform beneficial functions and these are known as plant-growth promoting rhizobacteria (PGPR) [5]. Some rhizospheric bacteria are then capable of penetrating the surface of the roots and colonizing the internal tissues of the root (a compartment known as the endorhizosphere [6]) and these are called endophytes. These bacterial endophytes overcome plant defences and establish themselves as permanent inhabitants of internal tissues without causing harm to the host plant [7]. It is believed that bacterial endophytes interact closely with the host having less competition for nutrients and living in a more protected environment [8]. 

The beneficial effects of plant microbiota include the increased nutrient availability (biofertilization) by fixing atmospheric nitrogen (N) and solubilizing the inorganic soil phosphorous (P) [9,10,11]. Additionally, they can exert chemical stimulation of plant growth and/or tolerance of the host to abiotic stress (phytostimulation) by producing plant hormones like indol-3-acetic acid (IAA) or enzymes like 1-aminocyclopropane-1-carboxylate (ACC) deaminase. The IAA increases root elongation, root exudates and plant biomass [12] and the ACC deaminase lowers the ethylene levels promoting plant growth [13]. Another beneficial effect is the ability to compete with or inhibit/antagonize potential pathogens, or reduce their effects (antagonism) by producing antimicrobial compounds [14]. Other indirect but important effects of endophytic colonization and lifestyle, reviewed in Reference [15], include the release of quorum sensing signals such as acyl-homoserine lactones (AHL) or its degradation by quorum quenching (QQ), cell motility (swimming and swarming), the exopolysaccharide (EPS) production and enzymatic lipolytic and proteolytic activities.

Several studies have focused on the isolation and identification of rice bacterial endophytes from different locations and varieties [16]. Furthermore, a metagenomic analysis of the rice endosphere provided clues about its composition and functions for the plant host [17] as well its dynamic changes [18]. More recently, an extensive isolation, identification and plant-growth promoting traits determination of large culturable collection of rice bacterial endophytes have been performed [19], providing further information on bacterial diversity in the rice endosphere. Although the composition of the endophytic microbiota of various plants is being studied [7,20,21], our knowledge of the endophytic bacterial ecology remains limited. In addition, most studies involving PGPR and endophytic bacteria are restricted to monostrain set-ups under laboratory conditions [22] and our understanding of the role of the microbial community on plant growth remains largely unexplored.

The main objective of this study is to provide additional data regarding the bacterial endophytic diversity of rice, as well as to isolate and characterize promising novel strains with plant beneficial traits. We hypothesize that the endophytic isolates that possess beneficial traits can promote the growth of rice plants in laboratory conditions. We also explored the reductionist approach of a simplified community as inoculum, an emerging approach that would help to harness the power of the plant microbiome [23]. In these studies, we have used two high-yield rice cultivars extensively grown in Venezuela in 2014 as starting material. To our knowledge, this is the first study of its kind performed with Venezuelan rice.

## 2. Materials and Methods

**Figure 1:**
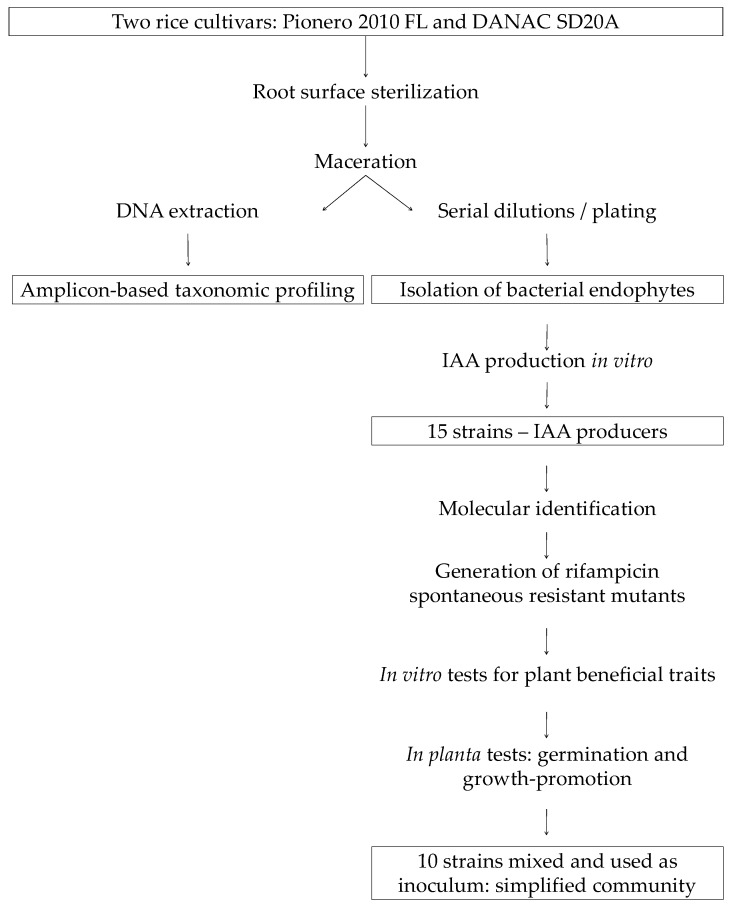
Methods workflow. Stepwise approach for determining the taxonomic profile of the bacterial endophytic microbiota of two rice cultivars and the setup of a simplified community based on in vitro and *in planta* performance of the isolates.

The outline of the methodology performed is shown in Figure 1. 

### 2.1. Sample Collection and Isolation of Bacteria

The rice plants were collected in April 2014 in the Paez Municipality, Portuguesa State, Venezuela. Three Pionero FL 2010 plants were sampled 88 days after planting from The Association of Certified Seed Producers of the Western Plains (APROSCELLO, for its acronym in Spanish) experimental plot. Three DANAC SD20A plants were sampled 90 days after planting from a private commercial plot. Each sampled healthy plant was randomly chosen and 5 m distant from each other. The sampled material was packaged in sterile bags and cooled at 4 °C for 4 days before bacterial isolation. Five grams of roots with the adherent soil were gently vortexed for 5 min in 20 mL of sterile saline solution (0.85% NaCl) and the rhizospheric soil suspensions were serially diluted and plated (100 µL) in triplicate on LB agar with cycloheximide (CHX) 50 mg/mL for determining the amount of rhizospheric colony-forming units (RCFU). The same 5 g of rice roots were then surface sterilized in 70% ethanol for 1 minute followed by 1.2% hypochlorite for 15 min with agitation and finally washed 6 times with sterile distilled water. The extent of the sterilization was verified by plating the final wash concentrated to 100 µL on LB plates before proceeding maceration. Sterilized roots were then macerated using sterile mortar and pestle in 10 mL of 0.85% NaCl sterile solution and different serial dilutions were plated in triplicate on LB/CHX plates for determining the number of putative endophytic colony-forming units (ECFU). The plates were incubated at 30 °C for 2 days. Independent ECFU showing distinct colony morphology were picked and streaked again on LB plates to ensure purity of the culture. The remnants of macerated roots and rhizospheric soil suspensions were then used for DNA extraction.

### 2.2. Total Bacterial Diversity of Rhizosphere and Endorhizosphere

The rhizospheric and endorhizospheric DNA from the two rice cultivars were extracted using Soilmaster DNA Extraction Kit (Epicentre, Madison, WI, USA) following the manufacturer’s guidelines. The quantity and quality of the DNA were assessed with Nanodrop (Thermo Fisher Scientific, Waltham, MA, USA) and electrophoresis in 0.7% agarose gel. The extracted DNA was then used as template for the first amplification of the V4 variable region of the 16S rRNA by PCR using primers 515F, 802R and 806R tailed with two different GC rich sequences enabling barcoding with a second amplification. Each sample was amplified in triplicate in 20 µL volume reactions containing 8 µL HotMasterMix 5Prime (Quanta Bio, Beverly, MA, USA), 0.4 µL BSA 20X, 1 µL EvaGreen™ 20X (Biotium, Fremont, CA, USA), 0.5 µL 515F primer (10 µM modified with unitail 1), 0.25 µL 802R primer (10 µM modified with unitail 2), 0.25 µL 806R primer (10 µM modified with unitail 2), 0.5 µL MitoBlk_515F V4 mitochondrial blocking primer (100 µM), 0.5 µL ChloBlk_806R V4 chloroplast blocking primer (100 µM and 2 µL (10–50 ng) of DNA template. The PCR amplifications were performed with CFX 96™ PCR System (Bio-Rad, Hercules, CA, USA) with 34 cycles of 94 °C for 20 s, 52 °C for 20 s, 65 °C for 40 s and a final extension of 65 °C for 2 min. The primary amplification takes advantage of rice specific V4 blocking mitochondrial and chloroplast primers in order to increase amplification of prokaryotic sequences. The rationale for these blocking PCR reactions has been previously described [24]. Deionized water was used in the negative controls.

The second PCR amplification (switch PCR) was required in order to attach the barcodes and was performed using a forward primer with the A adaptor (a sample-specific 10 bp barcode and the tail of the primary PCR primers) and a reverse primer with the P1 adaptor sequence and the reverse tail. The reaction was performed in 25 µL volume containing 10 µL HotMasterMix 5Prime, 1.25 µL EvaGreen™ 20X, 1.5 μL barcoded primer (10 µM), 1 μL of the first PCR product with the following conditions: 8 cycles of 94 °C for 10 s, 60 °C for 10 s, 65 °C for 40 s and a final extension of 72 °C for 3 min. The list of oligonucleotides used is shown in Supplementary Table S1.

We verified the size and the amount of the amplicons by agarose gel electrophoresis and were then pooled in equimolar amounts. The library was purified by the E-Gel^®^ SizeSelect™ (Invitrogen, Waltham, MA, USA) and verified the size and the amount with Agilent 2100 Bioanalyzer and a Qubit 1.0 fluorometer Q32857 (Thermo Fisher Scientific).

For sequencing, the library was submitted to emulsion PCR on the Ion OneTouch™ 2 system using the Ion PGM™ Template Hi-Q OT2 View (Life Technologies, Waltham, MA, USA) according to the manufacturer’s instructions. Ion sphere particles (ISP) were enriched using the E/S module. Resultant live ISPs were loaded and sequenced on an Ion 316 chip (Life Technologies). This sequencing was done in the Life Science Department of the University of Trieste (Trieste, Italy).

### 2.3. Plant-Growth Promoting Traits

Eighty-seven putative bacterial endophytes or EUFC were tested for indole-3-acetic acid (IAA) production in vitro. The bacterial cultures were grown in LB broth amended with tryptophan (100 µg/mL) at 30 °C for 4 days. The cells were sedimented by centrifugation and the supernatant (2 mL) was mixed with 4 mL of Salkowsky reagent (50 mL, 35% perchloric acid, 1 mL 0.5 M FeCl_3_ solution) and incubated in darkness for 30 min. The appearance of a red-pink colour indicated IAA production and OD_530nm_ was recorded [25]. The concentration of IAA produced by cultures was measured with a calibration graph of commercial IAA obtained in the range of 10–100 mg/mL and plotted in relation to the dry bacterial biomass. Fifteen bacterial isolates positive for the IAA production were chosen for further plant-growth promoting tests. Phosphate solubilisation was determined by growing bacteria on Pikovskaya agar as previously described [26]. The ACC deaminase activity was determined, as previously described in Reference [27], by comparing the growth of bacteria on minimal M9 medium without N source and M9 with 30 µmol of ACC as the sole N source. *N*-acyl homoserine lactone quorum sensing signal assays were carried out by using *Chromobacterium violaceum* CV026 and *C. violaceum* CV017 as biosensors [28]. Motility assay was performed as described previously [29]. The EPS production was assessed culturing the isolates on yeast extract mannitol medium as previously described [30]. The lipolytic activity was determined on 1/6 TSA medium amended with 1% tributyrin [31] and proteolytic activity on 1/6 TSA medium amended with 2% of powder milk [32]. The production of hydrogen cyanide (HCN) was estimated qualitatively as previously described [33]. The antibacterial activity against rice pathogens (*Dickeya zea*, *Pseudomonas fuscovaginae* and *Xanthomonas oryzae*) was carried out, plating the bacterial isolates on a bacterial lawn seeded with the pathogen.

### 2.4. Identification of Selected Isolates

Bacterial cells from 1 mL of overnight cultures were sedimented by centrifugation and resuspended in sterile PSB 0.5 mL. The cells were boiled for 3 min, cooled in ice 3 min and centrifuged at maximum speed for 5 min. The supernatants were used as a template in PCR reactions for amplifying 16S rDNA gene with the universal oligonucleotides fD1 and rP2 in 30 cycles of 95 °C 30 s, 57 °C 30 s and 72 °C 30 s with Taq DNA Polymerase (Promega, Madison, WI, USA). The PCR products were purified with EuroGOLD Gel Extraction Kit (EuroClone, Milan, Italy) following the manufacturer’s instructions and were sequenced with universal oligos 515F and 800R (Macrogen, Seoul, Korea) yielding > 1500 bp rDNA sequences. We identify the isolates by using EzBioCloud [34]. The phylogenetic analysis was performed on the Phylogeny online platform [35]. This software aligned the sequences with MUSCLE (v3.8.31), curated them with Gblocks (v0.91b), reconstructed the phylogenetic tree using the maximum likelihood method implemented in the PhyML program (v3.1/3.0 aLRT) and the tree rendering performed with TreeDyn (v198.3). 13 of 15 isolates were deposited in the Venezuelan Centre for Microorganisms Collection (Institute of Experimental Biology, Central University of Venezuela, Caracas, Venezuela) and the 16S rDNA sequences were deposited in GenBank (NCBI).

### 2.5. Seedling Early Growth, Endophytism and Plant-Growth Promotion Assay

In order to track endorhizosphere bacterial colonization after inoculation in gnotobiotic conditions, the generation of a rifampicin spontaneous resistant mutant was firstly achieved for the 15 selected isolates, as previously described [19,36]. The rifampicin resistance is commonly used as a marker to study survival kinetics of inoculated bacteria [37]. Single colonies of endophytic isolates were grown on 5 mL of LB medium for 24 h at 30 °C and aliquots of 100 uL were then plated on LB agar containing rifampicin (Rif) 100 µg/mL and incubated 48 h at 30 °C. Single rifampicin resistant colonies were re-streaked on LB Rif, stored at −80 °C and used for *in planta* experiments.

The rice seeds of the Baldo cultivar had a germination rate >97% (data not shown) and the effect of the bacterial inoculation on early growth was measured as the biomass of 4 days old seedlings. The seeds were surface sterilized for 30 min with 15% hypochlorite solution and then rinsed six times with sterile water. Fifty sterilized seeds were germinated in a Petri dish containing 20 mL sterilized water plus 500 µL of an overnight culture of each strain in 1 mL of LB medium, separately. The plates with seeds were kept in the dark at 30 °C for 4 days, before determining the wet weight of 10 groups of 5 germinated seeds, randomly chosen and with the water excess uniformly absorbed with clean paper. A control plate with only water (20 mL) and LB (500 µL) was included. Individual seedlings were then transferred to a 50 mL tube containing 35 mL of semisolid (0.25% agar) ½ Hoagland solution [38] and incubated at 28 °C, 75% humidity, 16 h/8 h light-dark cycles. The seedlings were watered every two days using ^1^/_10_ Hoagland solution. After 15 days, the inoculated plant roots were washed abundantly with tap water, dried with paper, separated from the aerial parts (cutting just below the cotyledon) and weighed. The root surface sterilization was performed as explained above and checked by plating the centrifuged sediment of the last wash (30 mL) on LB Rif 100 µg/mL. The roots were then macerated with sterile pestle and mortar with 3 mL of phosphate buffered saline (PBS) sterile solution and 100 µL of the macerate was plated on LB/Rif plates, incubated at 30 °C for 48 h. The CFU of recovered bacteria were counted and the number of the putative bacterial endophytes was calculated as CFU per gram of root. The aerial parts of the plants were dried at 65 °C for 5 days for determining the plant growth promotion. A control group of plants without bacteria was included. Five rice plants per treatment were harvested and processed. The mean of each treatment was compared to that in control with a two-tailed paired *t*-test (confidence interval 95%) using Graph Pad Prism version 5.0a (San Diego, CA, USA)

### 2.6. Simplified Community Colonization Assay

Ten bacterial strains were cultured for 48 h at room temperature in 10 mL of LB medium and diluted to OD_600nm_ of 2.0. The cells were then sedimented by centrifugation, washed with sterile 10 mL PBS and resuspended in 3 mL PBS. 2 mL of each bacterial/PBS suspension were mixed and finally, 30 mL of PBS were added bringing the final volume to 50 mL. 2 mL of this mixed suspension were used for DNA extraction and the remaining 48 mL were added to 800 mL of semisolid ½ Hoagland solution. A control without bacteria (only with LB broth) was included. One-week-old Baldo rice individual seedlings (sterilized and germinated as described above) were transferred to 40 mL (in Falcon tubes) of this community-containing semisolid Hoagland solution incubated and watered as described above. Three plants from the control and the treatment were recovered at 10, 20 and 30 days after planting, for a total of 18 plants harvested. The roots and aerial parts were separated and weighed. The roots were then sterilized and macerated with liquid nitrogen. The resulting root powder was used for DNA extraction and a 16S rRNA gene library was constructed and sequenced exactly as described in the section. 

### 2.7. Analyses of Sequencing Data

Reads were initially mapped against *O. sativa* mitochondrial (NC_011033) and plastidial genomes (NC_001320). Unmapped reads were further processed. We used CloVR 1.0 RC9 [39] on the Amazon Elastic Compute Cloud (EC2) to run the QIIME workflow “pick_otus_through_otu_tables.py” [40]. Within the QIIME workflow: (i) We set the minimum and maximum sequence length to 150 and 350 bp, respectively, the maximum homopolymer length to 8 bp and maximum number of ambiguous calls to zero; (ii) just after the quality filter we removed putative chimeras with UCHIME using the default parameters; (iii) clustering was performed using UCLUST with a nucleotide sequence identity threshold within each cluster at 97% and alignment against the Greengenes 16S database with PyNAST; (iv) taxonomy assignment of each OTU-representing sequence through the RDP classifier with a confidence threshold of 0.8 and (v) richness and diversity estimators (Chao, ACE, Simpson and Shannon) were computed by Mothur (alpha diversity) [41] and UniFrac (beta diversity) [42]. The effective number of species (ENS) was estimated using the conversion e^(Shannon)^ as proposed by [43].

## 3. Results

### 3.1. Bacterial Diversity of Venezuelan Rice Rhizosphere and Endorhizosphere

In order to obtain a picture of the taxonomic diversity of the two Venezuelan rice cultivars, the total rhizospheric and endorhizospheric bacterial community was assessed. After read-quality filtering, we obtained 326,496 high-quality bacterial reads with an average read length of 248 bp. The reads count per sample, as well as those obtained from plant organelles, are shown in Table 1. 

The blocking primer approach was effective in avoiding the amplification of plant-derived sequences when the bacterial populations were high. More than 99.8% of the queried sequences belonged to bacteria (Table 1A). A similar result was observed in the consortium-inoculated plants, with the 97.7% of reads belonging to bacteria 10 days after inoculation (Table 1B). However, the amplification block efficiency was lost in time. We also observed that in the control of uninoculated plants, the blocking PCR was not so efficient.

The snapshot of the total bacterial community showed a greater diversity of bacterial species in the rhizospheres than in the endorhizospheres, as supported by the diversity estimators (Table 2) combined with the rarefaction curve (Supplementary Figure S1). The rhizosphere of DANAC SD20A cultivar was colonized by a more abundant and diverse bacterial community than that of Pionero 2010 FL. The endorhizosphere of Pionero FL 2010 harboured a higher number of OTUs than that in DANAC SD20A. 

**Figure 2:**
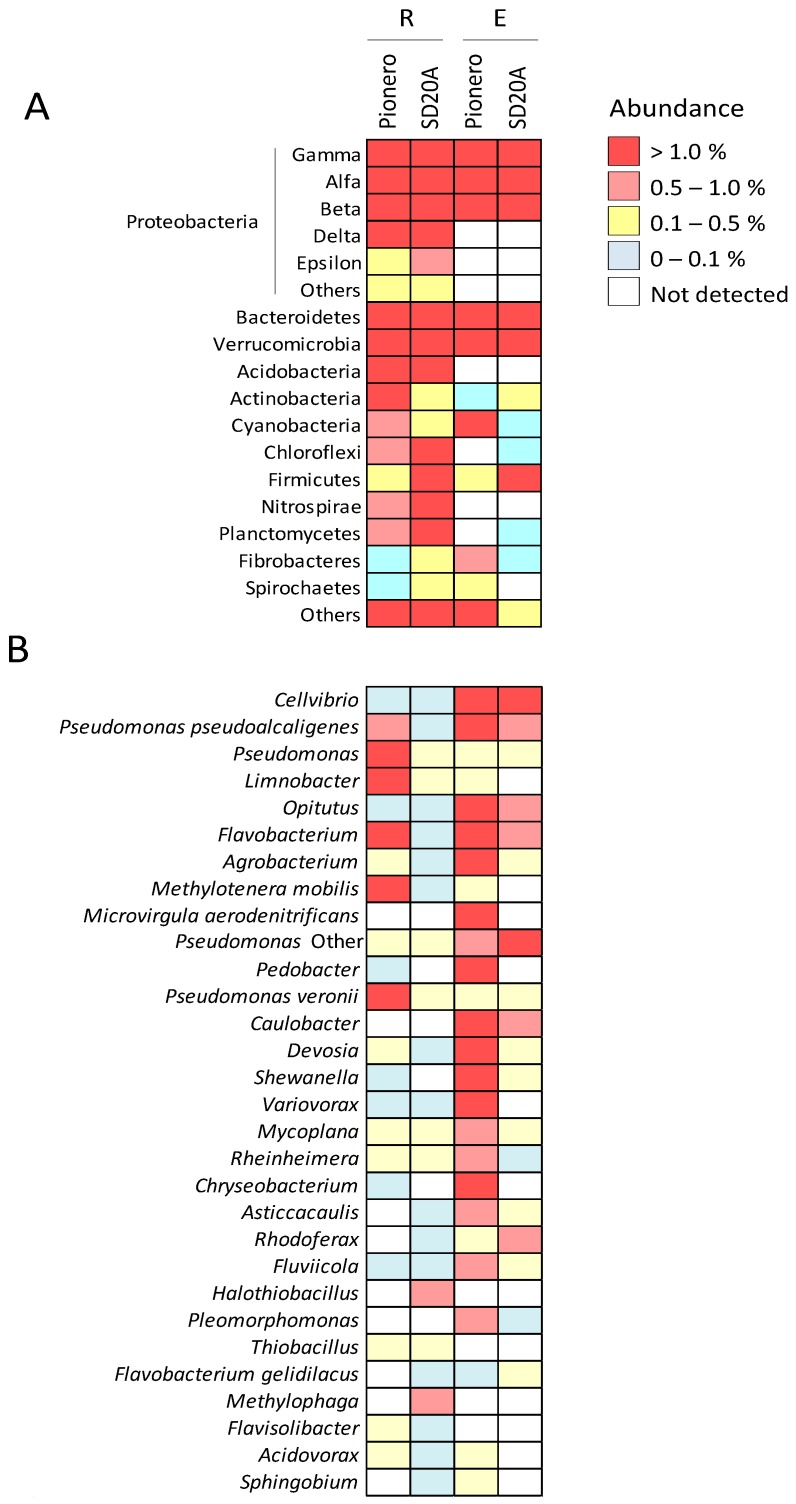
Bacterial microbiota analysis. Heat maps displaying the relative abundances of phyla and proteobacterial classes (**A**) and the most dominant genera or species (**B**), in the rhizosphere (R) and endorhizosphere (E) of both rice varieties.

After the removal of plant-derived, anonymous and singletons OTUs, the reads were clustered in a total of 341 different OTUs with a taxonomic assignment evaluated with >97% sequence identity as the cut-off. The complete lists of OTUs detected are shown in Supplementary Table S2 (relative abundances) and Supplementary Table S3 (number of reads). The distribution of OTUs and their abundance (expressed as the percentage on the total number of OTUs) at the phylum and proteobacterial classes level is summarized in Figure 2A. Proteobacteria dominated the bacterial microbiota, accounting for between 71% and 87% of the total OTUs. Among Proteobacteria, the Gammaproteobacteria class was the most abundant, followed by Betaproteobacteria and Alfaproteobacteria, while representatives of Deltaproteobacteria and Epsilonproteobacteria were not detected in the endorhizospheres. Bacteroidetes and Verrucomicrobia phyla were abundant across the samples. Acidobacteria and Nitrospirae representatives were only found in rhizospheric samples. Cyanobacteria phylum was enriched in Pionero FL 2010, while Firmicutes was enriched in DANAC SD20A cultivar.

The clustering of the 30 most abundant genera is shown in Figure 2B. They belong to three phyla: Proteobacteria (23), Bacteroidetes (6) and Verrucomicrobia (1). The following genera were dominant in the endorhizospheres: *Cellvibrio*, *Opitutus*, *Agrobacterium*, *Pedobacter*, *Devosia* and *Shewanella*. Interestingly, the *Caulobacter* genus was abundant and exclusively detected in both endorhizospheres. *Microvirgula* and *Pleomorfomonas* genera were also detected in endorhizospheres only, but with different distributions. The *Chryseobacterium* genus was associated with Pionero FL 2010 cultivar only.

### 3.2. Isolation of Culturable Bacteria from Rhizosphere and Endorhizosphere

The adherent soil of 5 g of roots (i.e. the rhizospheric soil) was serially diluted and plated in triplicate on LB/CHX plates. The average number of culturable bacteria was 5.5 × 10^7^ CFU per gram of rhizospheric soil. The surface-sterilized 5 g of macerated roots, on the other hand, yielded from 1420 to 361,120, with an average of 121,076 CFU/g. In order to perform the plant-growth promoting tests, 87 putative endophytic bacterial isolates were chosen based on colour and colony morphology differences.

### 3.3. Production of Indoleacetic Acid (IAA)

We tested the 87 putative bacterial endophytic isolates for the production of IAA, which is the main auxin in plants and an important phenotype linked to plant growth promotion. Thirty-five of the isolates were qualitative positive for IAA production, 17 from Pionero 2010 FL and 18 isolates from DANAC SD20A. Fifteen representative isolates were then chosen for further characterization namely: E1101, E1103, E1108, E1201, E1205, E1308, E2102, E2105, E2202, E2205, E2309, E2315, E2321, E2330 and E2330. In these isolates, the IAA production ranged from 0.153 to 4.86 µg/mg isolates (Supplementary Figure S2). 

### 3.4. Molecular Identification

**Figure 3:**
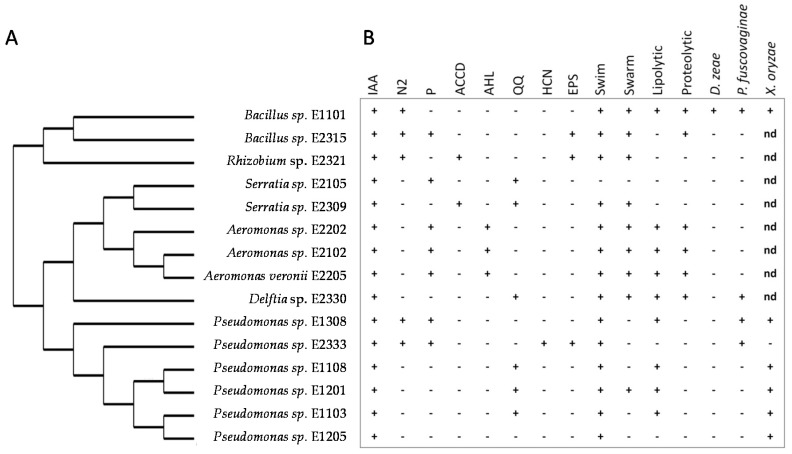
Putative endophytic bacteria isolated from surface-sterilized rice roots. (**A**) The bacterial isolates were identified by 16S sequencing and the rDNA sequences (average length 1518 bp) were used for constructing the cladogram. (**B**) Plant-growth promoting activities and antibacterial activities detected (+), non-detected (₋) or non-determined (nd) in in vitro tests. IAA, indole acetic acid production; N2, nitrogen fixation; P, phosphorous solubilisation; ACCD, ACC Deaminase activity; AHL, acyl homoserine lactone production; QQ, quorum quencher activity; HCN, hydrogen cyanide production; EPS, exopolysaccharide production; Swim and swarming and motility; Lipolytic and proteolytic activity; antibacterial activity against *Dickeya zea*, *Pseudomonas fuscovaginae* and *Xanthomonas oryzae*. The assays were performed in biological triplicates.

In order to identify and classify the 15 bacterial isolates which produced IAA, these isolates were subjected to 16S rDNA amplification and sequencing. The sequence analyses revealed that 2 isolates belong to the Firmicutes phylum (*Bacillus* sp. E1101 and *Bacillus* sp. E2315) and 13 to Proteobacteria. Of these, 1 belongs to Alfaproteobacteria (*Rhizobium* sp. E2321), 1 to Betaproteobacteria (*Delftia* sp. E2330) and 11 to Gammaproteobacteria (*Serratia* sp. E2105, *Serratia* sp. E2309; *Aeromonas* sp. E2102, *Aeromonas* sp. E2202; *Aeromonas veronii* E2205, *Pseudomonas* sp. E1201, *Pseudomonas* sp. E1103; *Pseudomonas* sp. E1108, *Pseudomonas* sp. E1205, *Pseudomonas E1308i* and *Pseudomonas* sp. E2333). The similarities with the closest type strain are shown in Table 3 and their phylogenetic relationships are shown in the cladogram in Figure 3A.

### 3.5. In Vitro Assays of Plant Beneficial Traits

It was of interest to determine whether the 15 IAA-producing putative rice bacterial endophytes possessed other important plant beneficial traits such as nitrogen fixation, phosphate solubilisation, ACC deaminase activity, HCN production and antibacterial activities. Other relevant traits for an endophytic lifestyle like quorum sensing acyl-homoserine lactone (AHL) production, quorum quenching activity, exopolysaccharide (EPS) production, motility and secretion of enzymes were also assayed. The results of these assays are summarized in Figure 3B.

### 3.6. Seedling Early Growth, Endophytism Assay and Plant-Growth Promotion

**Figure 4:**
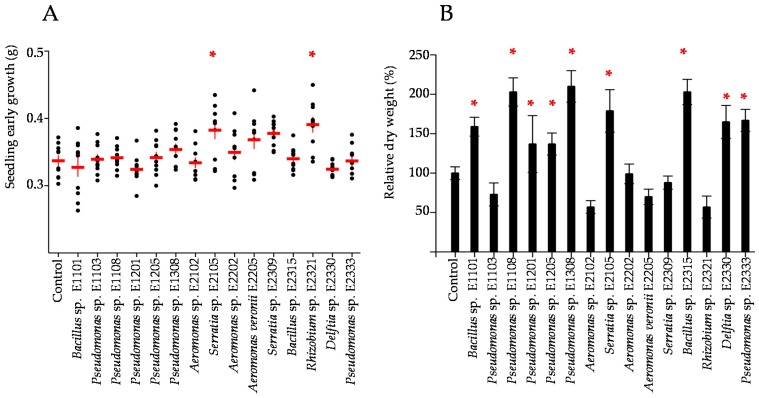
Plant growth promotion by single-strain inoculation. (**A**) Seedling early growth. Each dot represents the average wet weight of 5-days-old seedlings. The average and standard deviation are shown in red lines. (**B**) Plant growing rate. The dry weight of the aerial parts (stems and leaves) was determined. The averages are shown relative to the control (arbitrarily 100) with its standard deviation. The values were obtained from 5 different inoculated plants cultivated for 15 days. The red asterisks indicate statistical significance (*p* < 0.05).

Two strains significantly increased the seedling early growth. *Rhizobium* sp. E2321-germinated seeds were 7.6% heavier on average than control seeds and *Serratia* sp. E2309 with a 7.3% early growth increase (Figure 4A).

9 out of 15 isolates promoted significantly plant growth (Figure 4B). The dry weight of the plants inoculated with *Pseudomonas* sp. E1108, *Pseudomonas* sp. E1308 and *Bacillus* sp. E2315 doubled those of the control. Among them, *Pseudomonas* sp. E1308 was the best plant-growth promoter with an increase of 110% in dry weight when compared to the control (*p* < 0.05). The other 8 isolates showed increases of 103% (*Pseudomonas* sp. E1108 and *Bacillus* sp. E2315), 79% (*Serratia* sp. E2105), 67% (*Pseudomonas* sp. E2333), 65% (*Delftia* sp. E2330), 59% (*Bacillus* sp. E1101) and 37% (*Pseudomonas* sp. E1205 and E1201). Interestingly, only 1 isolate could be recovered from the endorhizosphere, namely *Pseudomonas* sp. E1308. The CFU of this isolate ranged from 170 to 44,000 CFU per gram of surface-sterilized roots.

### 3.7. Simplified Community Inoculation, Colonization and Plant Growth Promotion

**Figure 5:**
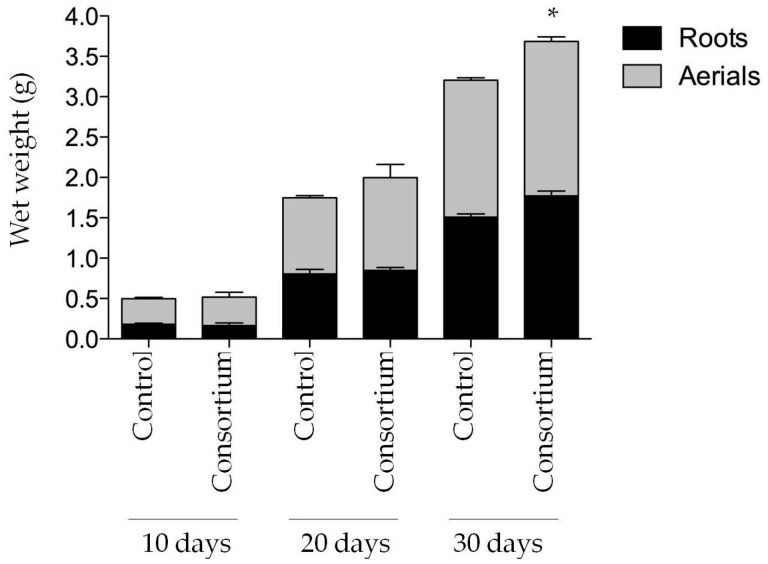
Effect of the bacterial consortium in plant growth. One-week-old rice seedlings were inoculated with a mixture of 10 bacterial strains and grown in controlled conditions for 30 days. Every ten days, 3 plants were harvested, cut in the two parts shown and weighted. A control without bacterial inoculation was included. The asterisk indicates statistical significance (*p* < 0.05).

It was of interest to perform *in planta* studies with a bacterial consortium in order to determine possible plant growth-promoting effects of a simplified community. We decided to use a bacterial consortium of 10 out of the 15 bacterial isolates, namely: *Pseudomonas* sp. E1108, *Pseudomonas* sp. E1205, *Pseudomonas* sp. E1308, *Aeromonas* sp. E2102, *A. veronii* E2205, *Serratia* sp. E2309, *Bacillus* sp. E2315, *Rhizobium* sp. E2321, *Delftia* sp. E2330 and *Pseudomonas* sp. E2333. An amount of bacterial suspension equivalent to OD_600nm_ of 2.0 of each culture was used for the mixed bacterial inoculum. This inoculum was included in the semisolid Hoagland solution where plants were grown. After 30 days, there was a significant increase of 15% (*p* < 0.05) in the wet weight of the inoculated plants compared to control non-inoculated, both in the roots and in aerial parts (Figure 5).

A cultivation-independent detection method using 16S rDNA amplicon sequencing was carried out in order to obtain insight into the colonization ability of the 10-strain simplified community over time. The numbers of reads obtained, bacterial- and plant-derived, are shown in Table 1 (B). Regarding the total bacterial endophytic abundance, it was noted that the uninoculated plants were systematically lower in bacterial populations at each time point compared to that in inoculated plants (Supplementary Figure S2).

**Figure 6:**
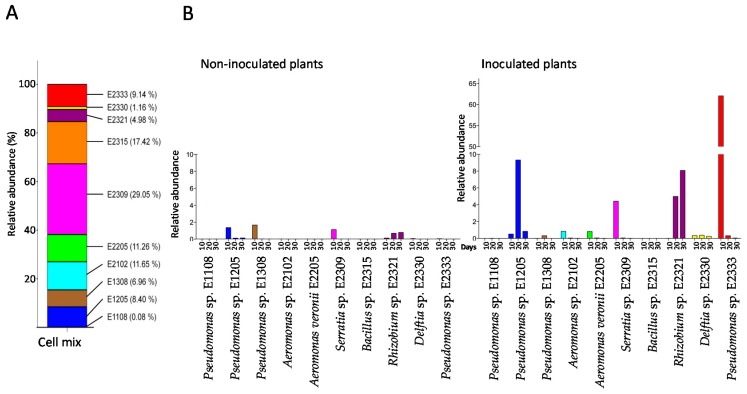
The composition of the 10-strains simplified community for 30 days growth of rice seedlings. (**A**) The cell mix represents the 10 species mixed and used as inoculum. The relative abundance of each strain is shown in brackets. The total number of reads was *n* = 45,246. (**B**) The relative abundance of each consortium strain was tracked at 10, 20 and 30 days after the inoculation of the rice seedlings. The results for non-inoculated and inoculated plants are shown in the coloured bars. The total number of reads was *n* = 111,291.

The composition of the bacterial cell mix (the pooled bacterial cultures that were then used as inoculum) varied from 36 reads (*Pseudomonas* sp. E1108) to 13,145 reads (*Serratia* sp. E2309) in a total of 45,246 reads, as shown in Figure 6A. In order to track the abundance of each strain of the bacterial consortium within the plants, their 16S sequences were used against the total 16S rDNA library sequenced. This was also performed for the control plants in order to determine if any seed-borne bacterial endophyte was taxonomically close enough to the strains used in the consortium, which could lead to false positives. The abundance of the simplified bacterial community was tracked in control and inoculated plants and it is represented as relative abundances in Figure 6B.

The abundance and identity of the reads suggested that taxonomically related strains to *Pseudomonas* sp. E1205, *Pseudomonas* sp. E1308, *Serratia* sp. E2309 and *Rhizobium* sp. E2321 were present in the control plants in low abundance. In the inoculated plants, at least 8 out of 10 bacterial strains were detected within the plant roots. Only 4 strains were however detected to some extent after 30 days of cultivation, namely: *Pseudomonas* sp. E1205, *Rhizobium* sp. E2321, *Delftia* sp. E2330 and *Pseudomonas* sp. E2333. This dataset suggested that these strains were capable to colonize together the rice roots.

## 4. Discussion

It is of great importance to study the microbiota diversity and functionality on the main agricultural crops [44], as well as to develop models for the study of plant-microbe interaction through simplified microbiota [45]. In this study, (i) we have performed a survey on the total bacterial endophytic community in *Oryza sativa* cv. Pionero FL 2010 and *O. sativa* cv. DANAC SD20A; (ii) we have carried out the isolation and partial characterization of 15 putative bacterial endophytes and (iii) we have narrowed a 4-strains simplified bacterial community as a starting point for a working model for bacteria-bacteria and bacteria-plant interactions in rice, towards a future efficient bioinoculant formulation possibly based on a mixed inoculum.

### 4.1. Amplicon-Based Taxonomic Profiling

The use of blocking primers was successful since >99.8% of the endorhizospheric reads belonged to bacteria. This is a substantial improvement in the obtaining of 16S rDNA libraries of endophytic bacteria, as it was already stated by [24] and when compared to our previous work [19]. We have noticed, however, that this approach should be optimized for samples with low bacterial DNA content.

Based on OTU analyses and diversity indices, the richness and species diversity were higher in the rhizospheres than in the endorhizospheres of both cultivars, an observation already widely documented [18,46,47]. Since most plant-associated bacteria are of soil origin [48] the largest bacterial diversity may be attributed to the primary site of interaction between plants and soil [49]. In addition, rhizospheres are rich in carbon sources secreted by plants [47], while endorhizospheres represent a relative stable compartment for microorganisms with less freely available carbon sources and other growth compounds.

Cluster analysis showed that Proteobacteria were by far the most predominant phylum in both compartments of both rice varieties and this is in agreement with several previous studies [17,18,19,50]. However, members of Deltaproteobacteria and Epsilonproteobacteria classes were not detected in the endorhizospheres analysed here; this is in contrast to what has been reported in a previous work of rice microbiome in Italy [15], Philippines [17] or China [51].

Among the top 30 genera, some show compartment specificity; for example, *Caulobacter*, *Microvirgula* and *Pleomorphomonas* were exclusively found in the endorhizospheres, while *Cellvibrio*, *Opitutus*, *Agrobacterium*, *Pedobacter*, *Devosia* and *Shewanella* were highly abundant in this compartment. The enrichment of certain microbes may indicate, as suggested by [18], that plants have the ability to select/recruit certain microbial consortia or that some microbes are more fit for endorhizosphere colonization.

### 4.2. Isolation of Putative Endophytic Bacteria, Determination of Its PGP Traits and Plant Colonization

Beneficial endophytic bacteria play important roles that positively affect directly or indirectly plant growth and development [52]. In this study, we selected 15 putative bacterial endophytes isolated from Venezuelan rice because they were IAA producers. IAA is the main auxin in plants, controlling root architecture, thereby improving nutrient acquisition [53,54,55]. Our estimations of the produced IAA are of milligrams of dry bacterial biomass rather than millilitres of culture since we think it could be more useful for future comparisons.

Two *Bacillus* strains, E1101 and E2315, were identified among our isolates. Although these two strains did not affect the early growth of rice seedlings, they positively influenced the plant growth after one month. Inoculation experiments, however, did not reveal them as endophytes. It is interesting to note that in our taxonomic profiling, *Bacilli* abundance was extremely low in the four compartments analysed, with a maximum abundance of 0.016% of the total reads. It cannot be excluded that the isolation procedure favoured the growth of *Bacillus* spp. or alternatively that the PCR for 16S-based taxonomic profiling was not so efficient for this bacterial group. 

The other 13 isolates belong to Proteobacteria, which is the most abundant phylum in the taxonomic analysis. *Rhizobium* sp. E2321 had the most positive impact on the seedling early growth rate. This strain displayed a number of PGP traits in vitro, however, it did not display beneficial effects *in planta*. This contradiction was discussed by Cardinale et al. [56] when they found similar discordance when analysing the effect of rhizobacteria on the growth of barley under salt stress. The isolate *Serratia* sp. E2309 was the only bacterial inoculum that increased the seedling early growth and plant growth. Others *Serratia* spp. have been previously reported as PGP strains [57,58,59] and therefore could be a good candidate for further studies. The other *Serratia* isolated (E2105) however did not result in any plant growth promotion. Our two *Serratia* isolates also displayed a different profile of in vitro activities, thus despite belonging to the same species, they possess considerable differences in their phenotypes. In our taxonomic profiling, *Serratia* spp. were not detected in the endorhizospheres of DANAC SD20A cultivar but were detected in low abundance in the rhizosphere of Pionero 2010 FL. This might be due to cultivation and/or PCR amplification bias.

Other isolates such as *Delftia* sp. E2330 and the others *Pseudomonas* spp. did not affect the seedling early growth but promoted the plant growth. In our taxonomic survey, *Delftia* spp. were present in low abundance in both compartments of DANAC SD20A cultivar. *Pseudomonas* spp. have been reported to be among the most abundant members of the rice endorhizospheres [16,17,60,61], thus it is not surprising that most of our isolates belong to this group. The *Aeromonas* spp. isolates did not show PGP activity in our study and this is in contrast with other studies on rice [62,63].

Of the 15 isolates, only *Pseudomonas* sp. E1308 could be re-isolated from the endosphere once inoculated; this is surprising and this could be due to their inability to enter the plant in the absence of other bacterial species (see below).

### 4.3. Seedling Inoculation with a Simplified Bacterial Community

Microorganisms do not act as individuals but rather act as a dynamically changing microbial community, where cells interact and communicate with one another. This communication influences bacterial behaviour, significantly affecting the phenotypes of the microbial community [64]. It is therefore of importance to developing new model systems for incorporating communities of microorganisms in plant microbiota research [45]. The use of traceable simplified ecosystems reduces the complexity of naturally complex microbiota and its investigation increase our knowledge regarding factors that shape and influence microbial communities. We therefore performed rice inoculations with a 10 strain simplified community in order to assess its potential for host colonization and possible differences compared to single strain inoculations. We did not include strains which possessed strong in vitro antibacterial activity (*Bacillus* sp. E1101) or putative redundant isolates (*Pseudomonas* sp. E1103 and E1201, *Serratia* sp. E2105 and *Aeromonas* sp. E2202). Assessing colonization via 16S rDNA gene community profiling showed that 8 strains were detected in the endorhizosphere. Within this group, *Pseudomonas* sp. E1205, *Rhizobium* sp. E2321, *Delftia* sp. E2330 and *Pseudomonas* sp. E2333 remained in the endorhizospheres after 30 days of plant growth. The isolate *Pseudomonas* sp. E1308, the only one recovered from surface-sterilized inoculated rice plants in the single-strain *in planta* tests, was surprisingly not detected when co-inoculated with the 9 other strains. The bacterial community can be influencing the endophytic colonization of this strain or the host plant favoured the colonization of the other strains. This finding could corroborate the knowledge of the compartment-mediated plant microbiota structure [18]. The design of simplified microbial communities has been recently considered as a priority for harnessing the plant microbiome in sustainable agriculture [45] and this approach has been addressed in *Arabidopsis* [65] and in maize [66]. 

### 4.4. Limitations of This Study

The results of this study clearly showed that the sampled plants harbour a bacterial microbiota differentially compartmentalized in their endorhizospheres. What is less clear is whether these differences are cultivar-specific. For overcoming this, the sampling procedure needs to be improved by including a larger number of samples. It must also be noted that all the amplicon-based taxonomic profiling are subjected to the intrinsic bias of the amplification, sequencing techniques and data processing [44], thus some taxa could not be appropriately represented. It has been reported that next generation sequencing technologies, like that used here, are prone to high errors rates [67]. 

For a better representation in the isolation of beneficial microbes, it would have been better if we used more than one culture medium. This would have a led to a more diverse set of bacterial species which would have benefited in the set-up of the mixed consortia; this is one of the major challenges of the simplified communities research [45]. In addition, in order to further optimize the bacterial mix in the consortia, the growth rates, metabolisms, antagonism of each member needs to be carefully assessed.

The putative endophytic strains were isolated from two rice cultivars which are genotypically different from the one used in the *in planta* experiments, hence it cannot be excluded that in our study plant genotype influences endorhizosphere colonization as suggested previously [45,50,68,69,70]. It is advisable to use the same cultivar for *in planta* studies as well as increasing the number of harvested plants in order to assess the rhizosphere and rhizoplane colonization. These limitations restrict our ability to make broader generalizations from our results. Nevertheless, the taxonomic range of putative bacterial endophytic microbiota of rice has been extended with this work and the isolated beneficial strains are good candidates for further experimentation.

## 5. Conclusions

This study provides an initial taxonomic survey of the bacterial rhizospheric and enorhizospheric microbiota of two Venezuelan rice varieties. The rice plants possessed a structured bacterial community indicating that there are bacterial taxa specifically associated to the rice endorhizosphere. Significant differences in the bacterial communities assembled in each cultivar were not highlighted by our experimental design; however, our results provide insight into the complex bacterial composition of the rice microbiota. Further studies are necessary to elucidate the rice core bacterial microbiome, the major species and their functional roles. Our initial simplified community results strongly encourage further studies of the synergistic, signalling and cooperative behaviour of multispecies consortia. 

## Figures and Tables

**Table 1:** Sequences characteristics. The number (#) and its corresponding percentage (%) of plant-derived and bacterial-derived 16S reads sequenced, as well as the average length in bp, are listed. (**A**) Results for the 16S-based taxonomic profiling of the two rice cultivars. (**B**) Results for the simplified community assay.

Samples		Plant Derived Reads		Bacterial Derived Reads		Average Length (bp)
		#	%	#	%	
**(A)** ***Amplicon-based taxonomic profiling***						
Pionero FL 2010	Rhizospheres	320	0.16	175,530	99.84	248
	Endorhizospheres	60	0.06	81,171	99.94	247
DANAC SD20A	Rhizospheres	81	0.09	49,374	99.91	249
	Endorhizospheres	16	0.04	20,421	99.96	248
**(B)** ***Simplified community***						
Control endorhizospheres	10 days	94,362	53.16	83,143	46.84	247
	20 days	111,238	95.63	5083	4.37	247
	30 days	153,205	98.41	2475	1.59	248
Inoculated endorhizospheres	10 days	3530	2.29	150,625	97.71	246
	20 days	100,128	57.54	73,887	42.46	248
	30 days	300,845	91.34	28,523	8.66	248

**Table 2:** Richness and diversity estimators. The observed diversity richness (OTUs), the OTUs richness (*Chao* and *ACE*) and diversity indexes (*Simpson*, *Shannon* and *ENS)* for the 16S rRNA libraries of the different samples.

		Richness Estimator			Diversity Estimator		
		OTUs	*Chao*	*ACE*	*Simpson*	*Shannon*	*ENS*
**Pionero FL2010**	**R**	1497	1549.6	1586.8	0.078	4.28	72
	**E**	794	825.5	855.2	0.089	3.74	42
**SD20A**	**R**	1620	1663.4	1706.7	0.014	5.52	250
	**E**	562	635.6	651.2	0.148	3.06	21

**Table 3:** Molecular identification of the putative bacterial endophytes isolated. The 16S rRNA genes were sequenced and compared to the prokaryotic database. The accession number to the NCBI (**A**), the accession number to the Venezuelan Centre for Microorganisms Collection (**B**), the closest type strain (**C**) and the corresponding reference sequence (**D**) are listed.

Rice Cultivar	Bacterial Isolate	Accession NCBI ^A^	AccessionCVCM ^B^	Closest Type Strain ^C^	Reference Sequence ^D^	Similarity
						(%)
Pionero 2010 FL	**E1101**	KY867521	CVCM2317	*Bacillus velezensis* CR-502^(T)^	AY603658	99.86
	**E1103**	KY867522	CVCM2318	*Pseudomonas gessardii* DSM 17152^(T)^	MNPU01000117	99.93
	**E1108**	KY867523	CVCM2319	*Pseudomonas chengduensis* MBR^(T)^	EU307111	99.93
	**E1201**	KY867525	CVCM2322	*Pseudomonas chengduensis* MBR^(T)^	EU307111	99.59
	**E1205**	KY867526	CVCM2324	*Pseudomonas oleovorans* subsp. *oleovorans* DSM 1045^(T)^	NIUB01000072	99.25
	**E1308**	KY867527	CVCM2326	*Pseudomonas gessardii* DSM 17152^(T)^	MNPU01000117	99.93
DANAC SD20A	**E2102**	KY867528	CVCM2328	*Aeromonas veronii* CECT 4257^(T)^	CDDK01000015	99.86
	**E2105**	KY867529	CVCM2329	*Serratia glossinae* C1^(T)^	FJ790328	99.66
	**E2202**	KY867530	-	*Aeromonas hydrophila* subsp. *hydrophila* ATCC 7966^(T)^	CP000462	99.93
	**E2205**	KY867531	CVCM2330	*Aeromonas veronii* CECT 4257^(T)^	CDDK01000015	100
	**E2309**	KY867532	CVCM2331	*Serratia glossinae* C1^(T)^	FJ790328	99.93
	**E2315**	KY867533	CVCM2334	*Bacillus altitudinis* 41KF2b^(T)^	ASJC01000029	99.93
	**E2321**	KY867534	CVCM2335	*Rhizobium oryziradicis* N19^(T)^	KX129901	98.08
	**E2330**	KY867535	-	*Delftia lacustris* LMG 24775^(T)^	jgi.1102360	99.93
	**E2333**	KY867536	CVCM2338	*Pseudomonas helmanticensis* OHA11^(T)^	HG940537	99.31

## Supplementary Materials

The following are available online at http://www.mdpi.com/2076-2607/6/1/14/s1.

Supplementary File 1

## References

[1] Food and Agriculture Organization of the United Nations (2013). FAO Statistical Yearbook 2013: World Food and Agriculture.

[2] Naher U.A., Othman R., Panhwar Q.A., Ismail M.R. (2015). Biofertilizer for sustainable rice production and reduction of environmental pollution. Crop Production and Global Environmental Issues.

[3] Singh J.S., Pandey V.C., Singh D.P. (2011). Efficient soil microorganisms: A new dimension for sustainable agriculture and environmental development. Agric. Ecosyst. Environ..

[4] Kim Y.C., Leveau J., Gardener B.B.M., Pierson E.A., Pierson L.S., Ryu C.M. (2011). The multifactorial basis for plant health promotion by plant-associated bacteria. Appl. Environ. Microbiol..

[5] Bhattacharyya P.N., Jha D.K. (2012). Plant growth-promoting rhizobacteria (PGPR): Emergence in agriculture. World J. Microbiol. Biotechnol..

[6] Rosenblueth M., Martínez-Romero E. (2006). Bacterial endophytes and their interactions with hosts. Mol. Plant Microbe Interact..

[7] Nair D.N., Padmavathy S. (2014). Impact of endophytic microorganisms on plants, environment and humans. Sci. World J..

[8] Reinhold-Hurek B., Hurek T. (2011). Living inside plants: Bacterial endophytes. Curr. Opin. Plant Biol..

[9] Pieterse C.M.J., Zamioudis C., Berendsen R.L., Weller D.M., Van Wees S.C., Bakker P.A. (2014). Induced Systemic Resistance by Beneficial Microbes. Annu. Rev. Phytopathol..

[10] Vacheron J., Desbrosses G., Bouffard M.L., Touraine B., Moenne-Loccoz Y., Muller D., Leqendre L., Wisniewski-Dye F., Prigent-Combaret C. (2013). Plant growth-promoting rhizobacteria and root system functioning. Front. Plant Sci..

[11] Lugtenberg B., Kamilova F. (2009). Plant-growth-promoting rhizobacteria. Annu. Rev. Microbiol..

[12] Etesami H., Alikhani H.A., Hosseini H.M. (2015). Indole-3-acetic acid (IAA) production trait, a useful screening to select endophytic and rhizosphere competent bacteria for rice growth promoting agents. MethodsX.

[13] Glick B.R. (2014). Bacteria with ACC deaminase can promote plant growth and help to feed the world. Microbiol. Res..

[14] Zdor R.E. (2015). Bacterial cyanogenesis: Impact on biotic interactions. J. Appl. Microbiol..

[15] Santoyo G., Moreno-Hagelsieb G., Mdel C.O., Glick B.R. (2016). Plant growth-promoting bacterial endophytes. Microbiol. Res..

[16] Mano H., Morisaki H. (2008). Endophytic bacteria in the rice plant. Microbes Environ..

[17] Sessitsch A., Hardoin P., Döring J., Weilharter A., Krause A., Woyke T., Mitter B., Hauberg-Lotte L., Friedrich F., Rahalkar M. (2012). Functional characteristics of an endophyte community colonizing rice roots as revealed by metagenomic analysis. Mol. Plant Microbe Interact..

[18] Edwards J., Johnson C., Santos-Medellín C., Lurie E., Podishetty N.K., Bhatnagar S., Eisen J.A., Sundaresan V. (2015). Structure, variation, and assembly of the root-associated microbiomes of rice. Proc. Natl. Acad. Sci. USA.

[19] Bertani I., Abbruscato P., Piffanelli P., Subramoni S., Venturi V. (2016). Rice bacterial endophytes: Isolation of a collection, identification of beneficial strains and microbiome analysis. Environ. Microbiol. Rep..

[20] Brader G., Compant S., Mitter B., Trognitz F., Sessitsch A. (2014). Metabolic potential of endophytic bacteria. Curr. Opin. Biotechnol..

[21] Marella S. (2014). Bacterial endophytes in sustainable crop production: Applications, recent developments and challenges ahead. Int. J. Life Sci. Res..

[22] Bulgarelli D., Schlaeppi K., Spaepen S., van Themaat E.V.L., Schulze-Lefert P. (2013). Structure and functions of the bacterial microbiota of plants. Annu. Rev. Plant Biol..

[23] Vorholt J.A., Vogel C., Carlström C.I., Müller D.B. (2017). Establishing Causality: Opportunities of Synthetic Communities for Plant Microbiome Research. Cell Host Microbe.

[24] Arenz B.E., Schlatter D.C., Bradeen J.M., Kinkel L.L. (2015). Blocking primers reduce co-amplification of plant DNA when studying bacterial endophyte communities. J. Microbiol. Methods.

[25] Bric J.M., Bostock R.M., Silverstone S.E. (1991). Rapid in situ assay for indoleacetic acid production by bacteria immobilized on a nitrocellulose membrane. Appl. Environ. Microbiol..

[26] Pikovskaya R. (1948). Mobilization of phosphorous in soil in connection with vital activity of some microbial species. Microbiologiya.

[27] Penrose D.M., Glick B.R. (2003). Methods for isolating and characterizing ACC deaminase-containing plant growth-promoting rhizobacteria. Physiol. Plant..

[28] McClean K.H., Winson M.K., Fish L., Tatlor A., Chhabra S.R., Camara M., Daykin M., Lamb J.H., Swift S., Bycroft B.W. (1997). Quorum sensing and Chromobacterium violaceum: Exploitation of violacein production and inhibition for the detection of *N*-acylhomoserine lactones. Microbiology.

[29] Kohler T., Curty L.K., Barja F., van Delden C., Pechere J.C. (2000). Swarming of Pseudomonas aeruginosa is dependent on cell-to-cell signaling and requires flagella and pili. J. Bacteriol..

[30] Zlosnik J.E.A., Hird T.J., Fraenkel M.C., Moreira L.M., Henry D.A., Speert D.P. (2008). Differential mucoid exopolysaccharide production by members of the *Burkholderia cepacia* complex. J. Clin. Microbiol..

[31] Smeltzer M.S., Hart M.E., Iandolo J.J. (1992). Quantitative spectrophotometric assay for staphylococcal lipase. Appl. Environ. Microbiol..

[32] Huber B., Riedel K., Hentzer M., Heydorn A., Gotschlich A., Givskov M., Molin S., Eberl L. (2001). The cep quorum-sensing system of *Burkholderia cepacia* H111 controls biofilm formation and swarming motility. Microbiology.

[33] Mehnaz S., Baig D.N., Lazarovits G. (2010). Genetic and Phenotypic Diversity of Plant Growth Promoting Rhizobacteria Isolated from Sugarcane Plants Growing in Pakistan. J. Microbiol. Biotechnol..

[34] Yoon S.H., Ha S.M., Kwon S., Lim J., Kim Y., Seo H., Chun J. (2017). Introducing EzBioCloud: A taxonomically united database of 16S rRNA gene sequences and whole-genome assemblies. Int. J. Syst. Evol. Microbiol..

[35] Dereeper A., Guiqnon V., Blanc G., Audic S., Buffet S., Chevenet F., Dufayard J.F., Guindon S., Ledort V., Lescot M. (2008). Phylogeny.fr: Robust phylogenetic analysis for the non-specialist. Nucleic Acids Res..

[36] Dey R., Pal K.K., Bhatt D.M., Chauhan S.M. (2004). Growth promotion and yield enhancement of peanut (*Arachis hypogaea* L.) by application of plant growth-promoting rhizobacteria. Microbiol. Res..

[37] Gamalero E., Lingua G., Berta G., Lemanceau P. (2009). Methods for studying root colonization by introduced beneficial bacteria. Sustainable Agriculture, 2009.

[38] Hoagland D.R., Arnon D.I. (1950). The Water-Culture Method for Growing Plants without Soil.

[39] Angiuoli S.V., Matalka M., Gussman A., Galens K., Vanqala M., Riley D.R., Arze C., White J.R., White O., Fricke W.F. (2011). CloVR: A virtual machine for automated and portable sequence analysis from the desktop using cloud computing. BMC Bioinform..

[40] Caporaso J.G., Kuczynski J., Stombauqh J., Bittinqer K., Bushman F.D., Costello E.K., Fierer N., Pena A.G., Goodrich J.K., Gordon J.I. (2010). QIIME allows analysis of high-throughput community sequencing data. Nat. Methods.

[41] Schloss P.D., Westcott S.L., Ryabin T., Hall J.R., Hartmann M., Hollister E.B., Lesniewski R.A., Oakley B.B., Parks D.H., Robins C.J. (2009). Introducing mothur: Open-source, platform-independent, community-supported software for describing and comparing microbial communities. Appl. Environ. Microbiol..

[42] Lozupone C., Lladser M.E., Knights D., Stombaugh J., Knight R. (2011). UniFrac: An effective distance metric for microbial community comparison. ISME J..

[43] Jost L., Devries P., Walla T., Greeney H., Chao A., Ricotta C. (2010). Partitioning diversity for conservation analyses. Divers. Distrib..

[44] Schlaeppi K., Bulgarelli D. (2015). The Plant Microbiome at Work. Mol. Plant Microbe Interact..

[45] Busby P.E., Soman C., Waqner M.R., Friesen M.L., Kremer J., Bennett A., Morsy M., Eisen J.A., Leach J.E., Danql J.L. (2017). Research priorities for harnessing plant microbiomes in sustainable agriculture. PLoS Biol..

[46] Hernández M., Dumont M.G., Yuan Q., Conrad R. (2015). Different bacterial populations associated with the roots and rhizosphere of rice incorporate plant-derived carbon. Appl. Environ. Microbiol..

[47] Mendes R., Garbeva P., Raaijmakers J.M. (2013). The rhizosphere microbiome: Significance of plant beneficial, plant pathogenic, and human pathogenic microorganisms. FEMS Microbiol. Rev..

[48] Hallmann J., Quadt-Hallmann A., Mahaffee W.F., Kloepper J.W. (1997). Bacterial endophytes in agricultural crops. Can. J. Microbiol..

[49] Hardoim P.R., Andreote F.D., Reinhold-Hurek B., Sessitsch A., van Overbeek L.S., van Elsas J.D. (2011). Rice root-associated bacteria: Insights into community structures across10 cultivars. FEMS Microbiol. Ecol..

[50] Santos-Medellín C., Edwards J., Liechty Z., Nguyen B., Sundaresan V. (2017). Drought stress results in a compartment-specific restructuring of the rice root-associated microbiomes. MBio.

[51] Sun L., Qiu F., Zhang X., Dai X., Dong X., Song W. (2008). Endophytic bacterial diversity in rice (*Oryza sativa* L.) roots estimated by 16S rDNA sequence analysis. Microb. Ecol..

[52] Müller D.B., Vogel C., Bai Y., Vorholt J.A. (2016). The Plant Microbiota: Systems-Level Insights and Perspectives. Annu. Rev. Genet..

[53] Duca D., Lorv J., Patten C.L., Rose D., Glick B.R. (2014). Indole-3-acetic acid in plant-microbe interactions. Antonie Van Leeuwenhoek.

[54] Sukumar P., Legué V., Vayssières A., Martin F., Tuskan G.A., Kalluri U.C. (2013). Involvement of auxin pathways in modulating root architecture during beneficial plant-microorganism interactions. Plant Cell Environ..

[55] Spaepen S., Vanderleyden J., Remans R. (2007). Indole-3-acetic acid in microbial and microorganism-plant signaling. FEMS Microbiol. Rev..

[56] Cardinale M., Ratering S., Suarez C., Montoya A.M.Z., Geissler-Plaum R., Schnell S. (2015). Paradox of plant growth promotion potential of rhizobacteria and their actual promotion effect on growth of barley (*Hordeum vulgare* L.) under salt stress. Microbiol. Res..

[57] Chakraborty U., Chakraborty B.N., Chakraborty A.P. (2010). Influence of Serratia marcescens TRS-1 on growth promotion and induction of resistance in Camellia sinensis against Fomes lamaoensis. J. Plant Interact..

[58] Neupane S., Hoqberq N., Alstrom S., Lucass S., Han J., Lapidus A., Chenq J.F., Bruce D., Goodqin L., Pitluck S. (2012). Complete genome sequence of the rapeseed plant-growth promoting Serratia plymuthica strain AS9. Stand. Genom. Sci..

[59] Devi U., Khatri I., Kuamr N., Kumar L., Sharma D., Subramanian S., Saini A.K. (2013). Draft Genome Sequence of a Plant Growth-Promoting Rhizobacterium, Serratia fonticola Strain AU-P3(3). Genome Announc..

[60] Prakamhang J., Minamisawa K., Teamtaisong K., Boonkerd N., Teaumroong N. (2009). The communities of endophytic diazotrophic bacteria in cultivated rice (*Oryza sativa* L.). Appl. Soil Ecol..

[61] Kaga H., Mano H., Tanaka F., Watanabe A., Kaneko S., Morisaki H. (2009). Rice Seeds as Sources of Endophytic Bacteria. Microbes Environ..

[62] Banik A., Mukhopadhaya S.K., Dangar T.K. (2016). Characterization of N_2_-fixing plant growth promoting endophytic and epiphytic bacterial community of Indian cultivated and wild rice (*Oryza* spp.) genotypes. Planta.

[63] Garcia-Cristobal J., Garcia-Villaraco A., Ramos B., Gutierrez-Mañero J., Lucas J.A. (2015). Priming of pathogenesis related-proteins and enzymes related to oxidative stress by plant growth promoting rhizobacteria on rice plants upon abiotic and biotic stress challenge. J. Plant Physiol..

[64] De Roy K., Marzorati M., van den Abbeele P., Van de Wiele T., Boon N. (2014). Synthetic microbial ecosystems: An exciting tool to understand and apply microbial communities. Environ. Microbiol..

[65] Bai Y., Muller D.B., Srinivas G., Garrido-Otyer R., Pptthoff E., Rott M., Dombrowski N., Munch P.C., Spaepen S., Remus-Emsermann M. (2015). Functional overlap of the Arabidopsis leaf and root microbiota. Nature.

[66] Niu B., Paulson J.N., Zheng X., Kolter R. (2017). Simplified and representative bacterial community of maize roots. Proc. Natl. Acad. Sci. USA.

[67] Knief C. (2014). Analysis of plant microbe interactions in the era of next generation sequencing technologies. Front. Plant Sci..

[68] Wagner M.R., Lundberg D.S., Del Rio T.G., Tringe S.G., Dangl J.L., Mitchell-Olds T. (2016). Host genotype and age shape the leaf and root microbiomes of a wild perennial plant. Nat. Commun..

[69] Patel J.S., Singh A., Singh H.B., Sarma B.K. (2015). Plant genotype, microbial recruitment and nutritional security. Front. Plant Sci..

[70] Turner T.R., James E.K., Poole P.S. (2013). The plant microbiome. Genome Biol..

