# Supplementary material for: Bacterial Microbiota of Rice Roots: 16S-Based Taxonomic Profiling of Endophytic and Rhizospheric Diversity, Endophytes Isolation and Simplified Endophytic Community
Source: Microorganisms. 2018 Feb 11;6(1):14. doi: 10.3390/microorganisms6010014 (PMC5874628; doi:10.3390/microorganisms6010014)
Supplement: Supplementary File 1 [file microorganisms-06-00014-s001.pdf]

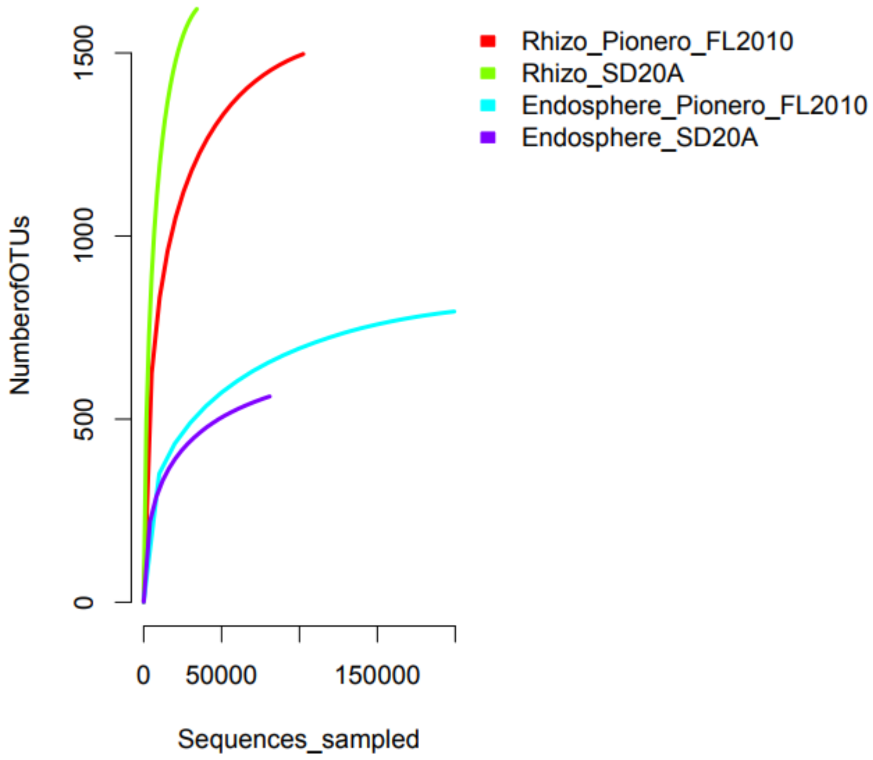

Supplementary Figure 1. Rarefaction curve. Representation of the observed number of OTUs as a function of sequences sampled.

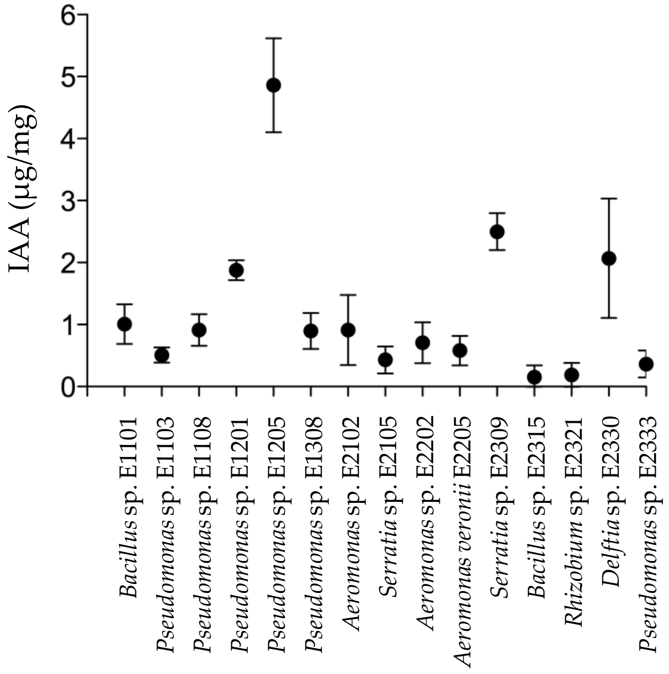

Supplementary Figure 2. Production of indole acetic acid (IAA) by the selected isolates. The 5 days old supernatant of each bacterial culture was spectrophotometrically analyzed after the Salvkoski reaction for the presence of IAA and the parallel construction of a calibration curve. Each dot represent de average reading of three replicates and the vertical bars the standard deviation. The values correspond to micrograms of IAA by milligram of dry bacterial biomass.

B

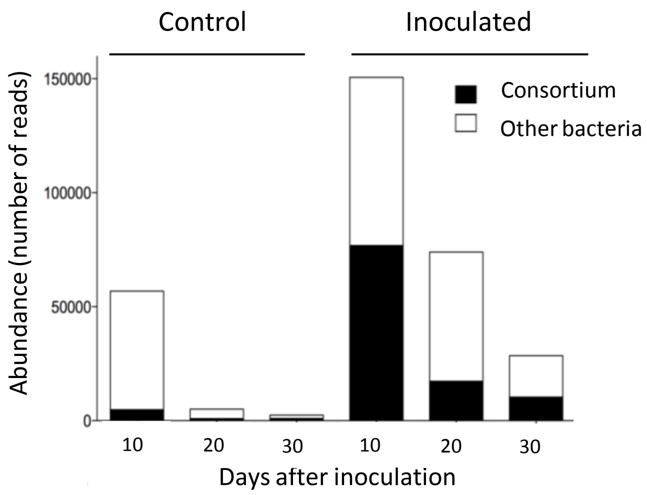

Supplementary Figure 3. 16S library sequencing in the simplified community assay. Distribution of the bacterial reads within the samples. The total bacterial reads is plotted for every group of samples and differentiated among those sequences matched with the 10 strains used as the inoculum (consortium) and those with no match with the consortium (other bacteria). The total numbers of reads were: for control plants 10 days n=177505; 20 days n=116321; 30 days n=155680. For inoculated plants 10 days n=154155; 20 days n=174015; 30 days n=329368 reads. Control plants refer to non-inoculated plants.

Supplementary Table 1. Oligonucleotide. In red, UNITAIL 1. In green, UNITAIL 2. A C3 phosphoramidite spacer was incorporated in the 3'-end (/3SpC3/) of the blocking primers. The 10 bp barcodes are underlined.

Name	Sequence 5' – 3'	Reference
First PCR round		
V4 515F	CAGGACCAGGGTACGGTGGTGCCAGCMGCCGCGGTAA	[71]
802R	CGCAGAGAGGCTCCGTGTACNVGGGTATCTAATCC	[72]
806R	CGCAGAGAGGCTCCGTGGACTACHVGGGTWTCTAAT	[71]
MitoBlk_515F	TCCCCATGCTTTCGCACCCCA**/3SpC3/**	This work
ChloBlk_806R	GTCTCTAATCCCATTTGCTCC**/3SpC3/**	This work
Second PCR round		
ION_UNI1_A_1	CCATCTCATCCCTGCGTGTCTCCGACTCAG**CTAAGGTAAC****CAGGACCAGGGTACGGTG**	This work
ION_UN1I_A_2	CCATCTCATCCCTGCGTGTCTCCGACTCAG**TAAGGAGAAC****CAGGACCAGGGTACGGTG**	This work
ION_UNI1_A_3	CCATCTCATCCCTGCGTGTCTCCGACTCAG**AAGAGGATTC****CAGGACCAGGGTACGGTG**	This work
ION_UNI1_A_4	CCATCTCATCCCTGCGTGTCTCCGACTCAG**TACCAAGATC****CAGGACCAGGGTACGGTG**	This work
ION_UNI1_A_5	CCATCTCATCCCTGCGTGTCTCCGACTCAG**CAGAAGGAAC****CAGGACCAGGGTACGGTG**	This work
ION_UNI1_A_6	CCATCTCATCCCTGCGTGTCTCCGACTCAG**CTGCAAGTTC****CAGGACCAGGGTACGGTG**	This work
ION_UNI1_A_7	CCATCTCATCCCTGCGTGTCTCCGACTCAG**TTCGTGATTC****CAGGACCAGGGTACGGTG**	This work
ION_UNI1_A_8	CCATCTCATCCCTGCGTGTCTCCGACTCAG**TTCCGATAAC****CAGGACCAGGGTACGGTG**	This work
ION_UNI1_A_9	CCATCTCATCCCTGCGTGTCTCCGACTCAG**TGAGCGGAAC****CAGGACCAGGGTACGGTG**	This work
ION_UNI1_A_10	CCATCTCATCCCTGCGTGTCTCCGACTCAG**CTGACCGAAC****CAGGACCAGGGTACGGTG**	This work
ION_UNI1_A_11	CCATCTCATCCCTGCGTGTCTCCGACTCAG**TCCTCGAATC****CAGGACCAGGGTACGGTG**	This work
ION_UNI1_A_12	CCATCTCATCCCTGCGTGTCTCCGACTCAG**TAGGTGGTTC****CAGGACCAGGGTACGGTG**	This work
ION_UNI1_A_13	CCATCTCATCCCTGCGTGTCTCCGACTCAG**TCTAACGGAC****CAGGACCAGGGTACGGTG**	This work
ION_UNI1_A_14	CCATCTCATCCCTGCGTGTCTCCGACTCAG**TTGGAGTGTC****CAGGACCAGGGTACGGTG**	This work
ION_UNI1_A_15	CCATCTCATCCCTGCGTGTCTCCGACTCAG**TCTAGAGGTC****CAGGACCAGGGTACGGTG**	This work
ION_UNI1_A_16	CCATCTCATCCCTGCGTGTCTCCGACTCAG**TCTGGATGAC****CAGGACCAGGGTACGGTG**	This work
ION_UNI1_A_17	CCATCTCATCCCTGCGTGTCTCCGACTCAG**TCTATTCGTC****CAGGACCAGGGTACGGTG**	This work
ION_UNI1_A_18	CCATCTCATCCCTGCGTGTCTCCGACTCAG**AGGCAATTGC****CAGGACCAGGGTACGGTG**	This work
ION_UNI1_A_19	CCATCTCATCCCTGCGTGTCTCCGACTCAG**TTAGTCGGAC****CAGGACCAGGGTACGGTG**	This work
ION_UNI1_A_20	CCATCTCATCCCTGCGTGTCTCCGACTCAG**CAGATCCATC****CAGGACCAGGGTACGGTG**	This work
ION_UNI1_A_21	CCATCTCATCCCTGCGTGTCTCCGACTCAG**TCGCAATTAC****CAGGACCAGGGTACGGTG**	This work
ION_UNI1_A_22	CCATCTCATCCCTGCGTGTCTCCGACTCAG**TTCGAGACGC****CAGGACCAGGGTACGGTG**	This work
ION_UNI1_A_23	CCATCTCATCCCTGCGTGTCTCCGACTCAG**TGCCACGAAC****CAGGACCAGGGTACGGTG**	This work
ION_UNI1_A_24	CCATCTCATCCCTGCGTGTCTCCGACTCAG**AACCTCATTC****CAGGACCAGGGTACGGTG**	This work
ION_UNI_trP1 Rev	CCTCTCTATGGGCAGTCGGTGATCGCAGAGAGGCTCCGTG	This work
**For 16S sequencing**		
fD1	AGAGTTTGATCCTGGCTCAG	Universal
rP2	ACGGCTACCTTGTTACGACTT	Universal
518F	CCAGCAGCCGCGGTAATACG	Universal
800R	TACCAGGGTATCTAATCC	Universal

Supplementary table 2. List of bacterial genera identified. For the rhizospheres (R) of Pionero 2010 FL (Pionero) and DANAC SD20A (SD20A) had 175530 and 49374 reads respectively. The endorhizospheres (E) had 81171 and 49374 reads, respectively.

	Genus	R	R	E	E
		Pionero	SD20A	Pionero	SD20A
1	g___s__	0.018	0.000	0.000	0.000
2	g__4-29_s__	0.009	0.057	0.000	0.000
3	g__A17_s__	0.003	0.000	0.000	0.000
4	g__Abiotrophia_s__	0.000	0.001	0.000	0.000
5	g__Achromobacter_s__	0.003	0.000	0.084	0.009
6	g__Acidovorax_Other	0.002	0.000	0.003	0.000
7	g__Acidovorax_s__	0.256	0.033	0.165	0.000
8	g__Acidovorax_s__delafieldii	0.004	0.000	0.002	0.000
9	g__Acidovorax_s__facilis	0.000	0.000	0.001	0.000
10	g__Acinetobacter_s__	0.036	0.002	0.122	0.087
11	g__Acinetobacter_s__johnsonii	0.000	0.002	0.000	0.000
12	g__Acinetobacter_s__lwoffii	0.004	0.000	0.030	0.103
13	g__Acinetobacter_s__rhizosphaerae	0.015	0.000	0.000	0.000
14	g__Actinobacillus_Other	0.001	0.003	0.000	0.000
15	g__Actinotalea_s__	0.000	0.000	0.000	0.005
16	g__Adhaeribacter_s__	0.002	0.026	0.000	0.000
17	g__Aeromonas_Other	0.000	0.000	0.000	0.000
18	g__Aeromonas_s__caviae	0.001	0.000	0.011	0.002
19	g__Aggregatibacter_s__	0.000	0.002	0.000	0.031
20	g__Agrobacterium_Other	0.000	0.000	0.002	0.000
21	g__Agrobacterium_s__	0.179	0.077	2.459	0.472
22	g__Agrobacterium_s__undicola	0.000	0.000	0.239	0.000
23	g__Agrobacterium_s__vitis	0.000	0.000	0.001	0.000
24	g__Alcanivorax_s__	0.000	0.001	0.000	0.000
25	g__Algoriphagus_s__terrigena	0.000	0.079	0.000	0.221
26	g__Amaricoccus_s__	0.002	0.000	0.000	0.003
27	g__Aminobacter_s__	0.000	0.007	0.000	0.000
28	g__Amorphomonas_s__oryzae	0.000	0.001	0.000	0.000
29	g__Anaerococcus_s__	0.000	0.004	0.000	0.000
30	g__Anaerolinea_s__	0.005	0.006	0.000	0.000
31	g__Anaeromyxobacter_s__	0.080	0.094	0.000	0.000
32	g__Anaerovorax_s__	0.000	0.000	0.000	0.001
33	g__Ancylobacter_s__	0.033	0.000	0.000	0.007
34	g__Antarctobacter_s__	0.000	0.000	0.000	0.005
35	g__Aquaspirillum_s__putridiconchylium	0.000	0.031	0.000	0.000
36	g__Aquicella_s__	0.005	0.002	0.000	0.000
37	g__Aquimarina_s__	0.000	0.000	0.000	0.020
38	g__Aquimonas_s__	0.000	0.001	0.000	0.000
39	g__Arenimonas_s__	0.047	0.381	0.000	0.000
40	g__Arthrobacter_Other	0.005	0.000	0.000	0.000
41	g__Arthrobacter_s__psychrolactophilus	0.029	0.000	0.000	0.000
42	g__Arthronema_s__	0.000	0.004	0.000	0.000
43	g__Aspromonas_s__composti	0.000	0.001	0.000	0.000
44	g__Asticcacaulis_Other	0.000	0.000	0.001	0.000
45	g__Asticcacaulis_s__	0.000	0.007	0.515	0.477
46	g__Azospira_s__	0.000	0.001	0.000	0.000
47	g__Azospirillum_s__	0.000	0.001	0.205	0.008
48	g__Azospirillum_s__massiliensis	0.000	0.000	0.326	0.119

49	g__Bacillus_Other	0.000	0.000	0.018	0.000
50	g__Bacillus_s__	0.002	0.001	0.000	0.001
51	g__Bacillus_s__cereus	0.009	0.001	0.000	0.000
52	g__Bacteroides_s__	0.000	0.004	0.000	0.000
53	g__Bdellovibrio_s__	0.012	0.005	0.000	0.000
54	g__Bdellovibrio_s__bacteriovorus	0.000	0.001	0.000	0.000
55	g__Blastomonas_s__	0.014	0.002	0.000	0.000
56	g__Blvii28_s__	0.000	0.001	0.000	0.000
57	g__Bosea_s__genosp.	0.000	0.000	0.045	0.002
58	g__Bradyrhizobium_s__	0.001	0.000	0.001	0.000
59	g__Brevibacillus_s__	0.000	0.000	0.015	0.000
60	g__Brevibacterium_s__aureum	0.002	0.000	0.000	0.001
61	g__Brevundimonas_Other	0.000	0.000	0.000	0.000
62	g__Brevundimonas_s__diminuta	0.000	0.004	0.047	0.000
63	g__Bulleidia_s__moorei	0.000	0.000	0.000	0.006
64	g__Burkholderia_s__	0.000	0.000	0.000	0.000
65	g__Candidatus Endobugula_s__	0.000	0.000	0.000	0.003
66	g__Candidatus Koribacter_s__	0.076	0.022	0.000	0.000
67	g__Candidatus Nitrososphaera_s__SCA1170	0.027	0.005	0.000	0.000
68	g__Candidatus Rhabdochlamydia_s__	0.001	0.000	0.030	0.000
69	g__Candidatus Solibacter_s__	0.026	0.010	0.000	0.000
70	g__Candidatus Xiphinematobacter_s__	0.020	0.001	0.050	0.000
71	g__Capnocytophaga_s__	0.000	0.000	0.000	0.003
72	g__Capnocytophaga_s__ochracea	0.000	0.001	0.000	0.000
73	g__Catonella_s__	0.000	0.000	0.000	0.002
74	g__Caulobacter_Other	0.000	0.000	0.033	0.000
75	g__Caulobacter_s__	0.000	0.001	1.224	0.923
76	g__Cellulomonas_s__	0.000	0.000	0.000	0.002
77	g__Cellvibrio_s__	0.013	0.042	14.755	5.257
78	g__Chelativorans_s__	0.001	0.000	0.000	0.000
79	g__Chryseobacterium_s__	0.023	0.002	1.093	0.000
80	g__Citrobacter_s__	0.000	0.000	0.000	0.072
81	g__Cloacibacterium_s__	0.075	0.031	0.013	0.000
82	g__Clostridium_Other	0.000	0.001	0.000	0.000
83	g__Clostridium_s__	0.000	0.002	0.000	0.000
84	g__Clostridium_s__acetobutylicum	0.000	0.002	0.000	0.000
85	g__Clostridium_s__butyricum	0.004	0.000	0.000	0.000
86	g__Clostridium_s__hungatei	0.000	0.002	0.000	0.000
87	g__Clostridium_s__intestinale	0.005	0.000	0.000	0.000
88	g__Coccinimonas_s__marina	0.014	0.000	0.000	0.000
89	g__Cohnella_s__	0.000	0.000	0.000	0.009
90	g__Comamonas_s__	0.024	0.000	0.000	0.000
91	g__Constrictibacter_s__antarcticus	0.000	0.002	0.000	0.000
92	g__Coprococcus_s__	0.000	0.000	0.000	0.002
93	g__Corynebacterium_s__	0.021	0.003	0.002	0.037
94	g__Corynebacterium_s__kroppenstedtii	0.001	0.001	0.000	0.000
95	g__Crenothrix_s__	0.000	0.001	0.000	0.000
96	g__Crocinitomix_s__	0.000	0.000	0.052	0.000
97	g__Cryocola_s__	0.000	0.000	0.010	0.000
98	g__Cylindrospermopsis_s__	0.000	0.002	0.000	0.000

99	g__Cytophaga_s__	0.023	0.000	0.000	0.000
100	g__DA101_s__	0.010	0.000	0.000	0.000
101	g__DCE29_s__	0.003	0.000	0.000	0.000
102	g__Dechloromonas_s__	0.026	0.000	0.000	0.000
103	g__Defluviitalea_s__saccharophila	0.012	0.000	0.000	0.000
104	g__Delftia_s__	0.002	0.013	0.000	0.010
105	g__Demequina_s__	0.000	0.000	0.000	0.001
106	g__Desulfobacca_s__	0.000	0.017	0.000	0.000
107	g__Desulfobulbus_s__	0.013	0.012	0.000	0.000
108	g__Desulfococcus_s__	0.000	0.010	0.000	0.000
109	g__Desulfomicrobium_s__	0.000	0.001	0.000	0.000
110	g__Desulfomonile_s__	0.000	0.004	0.000	0.000
111	g__Desulforhabdus_s__amnigena	0.001	0.000	0.000	0.000
112	g__Desulfotalea_s__	0.000	0.002	0.000	0.000
113	g__Desulfovibrio_s__	0.002	0.006	0.000	0.000
114	g__Desulfovibrio_s__mexicanus	0.000	0.002	0.000	0.000
115	g__Desulfovibrio_s__putealis	0.010	0.002	0.000	0.000
116	g__Desulfovirga_s__adipica	0.004	0.006	0.000	0.000
117	g__Devosia_s__	0.241	0.057	1.504	0.117
118	g__Dok59_s__	0.007	0.005	0.000	0.000
119	g__Dokdonella_s__	0.011	0.002	0.009	0.000
120	g__Dyadobacter_s__	0.162	0.002	0.012	0.000
121	g__Eikenella_s__	0.000	0.001	0.000	0.008
122	g__Endozoicomonas_s__montiporae	0.000	0.000	0.001	0.000
123	g__Enhydrobacter_s__	0.018	0.008	0.070	0.180
124	g__Enterobacter_s__	0.000	0.000	0.001	0.000
125	g__Epulopiscium_s__	0.012	0.000	0.000	0.000
126	g__Erythrobacter_Other	0.001	0.000	0.000	0.000
127	g__Erythrobacter_s__	0.058	0.000	0.000	0.000
128	g__Escherichia_s__coli	0.026	0.000	0.033	0.005
129	g__Euptelea_s__polyandra	0.000	0.000	0.000	0.000
130	g__Exiguobacterium_s__	0.005	0.000	0.031	0.000
131	g__Fimbriimonas_s__	0.010	0.003	0.000	0.000
132	g__Flavisolibacter_s__	0.421	0.070	0.000	0.000
133	g__Flavobacterium_Other	0.002	0.000	0.026	0.000
134	g__Flavobacterium_s__	1.669	0.039	1.483	0.585
135	g__Flavobacterium_s__frigidarium	0.000	0.000	0.000	0.001
136	g__Flavobacterium_s__gelidilacus	0.000	0.069	0.097	0.365
137	g__Flavobacterium_s__succinicans	0.012	0.021	0.179	0.181
138	g__Flectobacillus_s__	0.000	0.001	0.000	0.000
139	g__Fluviicola_s__	0.021	0.012	0.559	0.214
140	g__Francisella_s__	0.001	0.000	0.000	0.000
141	g__Fritschea_s__eriococci	0.001	0.000	0.000	0.000
142	g__Fusibacter_s__	0.010	0.003	0.000	0.000
143	g__Fusobacterium_s__	0.000	0.000	0.005	0.024
144	g__Gallionella_s__	0.020	0.067	0.000	0.000
145	g__Gemmatimonas_s__	0.000	0.002	0.000	0.000
146	g__Geobacter_s__	0.146	0.050	0.000	0.000
147	g__GOUTA19_s__	0.096	0.059	0.000	0.000
148	g__Granulicatella_s__	0.000	0.003	0.000	0.002

149	g__Haemophilus_s__parainfluenzae	0.002	0.016	0.000	0.002
150	g__Halomonas_s__	0.000	0.024	0.000	0.053
151	g__Halothiobacillus_s__	0.000	0.675	0.000	0.000
152	g__Herbaspirillum_s__	0.000	0.000	0.024	0.001
153	g__HTCC_s__	0.000	0.012	0.000	0.000
154	g__Hydrogenophaga_s__	0.085	0.033	0.012	0.009
155	g__Hylemonella_s__	0.002	0.000	0.000	0.000
156	g__Hymenobacter_s__	0.002	0.000	0.000	0.000
157	g__Hyphomicrobium_Other	0.000	0.001	0.000	0.000
158	g__Hyphomicrobium_s__	0.017	0.008	0.000	0.000
159	g__Hyphomonas_s__	0.000	0.003	0.000	0.000
160	g__Iamia_s__	0.000	0.002	0.000	0.000
161	g__Janthinobacterium_s__	0.007	0.000	0.003	0.005
162	g__Janthinobacterium_s__lividum	0.024	0.000	0.030	0.000
163	g__K82_s__	0.000	0.001	0.000	0.000
164	g__Kaistia_s__	0.000	0.002	0.000	0.002
165	g__Kaistobacter_s__	0.327	0.117	0.000	0.000
166	g__Klebsiella_s__	0.000	0.002	0.021	0.000
167	g__Kocuria_s__rhizophila	0.000	0.000	0.000	0.002
168	g__Lacibacter_s__cauensis	0.104	0.041	0.000	0.018
169	g__Lactobacillus_s__zeae	0.000	0.000	0.006	0.000
170	g__LCP-6_s__	0.015	0.036	0.000	0.000
171	g__Leadbetterella_s__	0.018	0.028	0.000	0.000
172	g__Leptolyngbya_s__	0.000	0.003	0.000	0.000
173	g__Leptonema_s__	0.000	0.002	0.000	0.000
174	g__Leptospira_s__	0.004	0.003	0.000	0.000
175	g__Leptotrichia_s__	0.000	0.000	0.010	0.000
176	g__Leuconostoc_s__	0.000	0.000	0.000	0.001
177	g__Limnobacter_s__	5.641	0.319	0.263	0.000
178	g__Limnohabitans_s__	0.001	0.001	0.006	0.000
179	g__Loktanella_s__	0.000	0.000	0.010	0.002
180	g__Luteimonas_s__	0.012	0.003	0.000	0.000
181	g__Luteolibacter_s__	0.006	0.002	0.168	0.001
182	g__Lutibacterium_s__	0.000	0.000	0.241	0.000
183	g__Lutimonas_s__	0.000	0.001	0.000	0.007
184	g__Lysobacter_s__	0.003	0.005	0.058	0.011
185	g__Magnetospirillum_s__	0.000	0.000	0.000	0.003
186	g__Maribacter_s__	0.001	0.000	0.000	0.000
187	g__Marinobacter_s__	0.215	0.000	0.000	0.000
188	g__Marinobacter_s__bryozoorum	0.001	0.000	0.000	0.000
189	g__Massilia_s__haematophila	0.001	0.000	0.000	0.000
190	g__Mesorhizobium_s__	0.000	0.004	0.000	0.000
191	g__Methylibium_s__	0.006	0.005	0.000	0.000
192	g__Methylobacterium_s__	0.006	0.001	0.000	0.000
193	g__Methylomicrobium_s__	0.000	0.002	0.000	0.000
194	g__Methylomicrobium_s__agile	0.000	0.011	0.000	0.000
195	g__Methylophaga_s__	0.000	0.511	0.000	0.000
196	g__Methylotenera_s__mobilis	2.872	0.028	0.150	0.000
197	g__Methyloversatilis_s__	0.039	0.053	0.002	0.006
198	g__Methylovorus_s__glucosotrophus	0.000	0.000	0.047	0.000

199	g__Micrococcus_s__	0.000	0.000	0.000	0.001
200	g__Microvirgula_s__aerodenitrificans	0.000	0.001	2.514	0.000
201	g__Muricola_s__jejuensis	0.000	0.000	0.013	0.000
202	g__Mycoplana_s__	0.227	0.241	0.889	0.229
203	g__Mycoplasma_s__	0.000	0.001	0.000	0.000
204	g__Myxococcus_s__	0.000	0.006	0.000	0.000
205	g__Nautella_s__	0.000	0.000	0.005	0.000
206	g__Neisseria_s__	0.017	0.004	0.000	0.011
207	g__Neisseria_s__oralis	0.000	0.001	0.000	0.000
208	g__Neisseria_s__subflava	0.000	0.015	0.000	0.007
209	g__Nevskia_s__ramosa	0.069	0.012	0.261	0.026
210	g__Niabella_s__	0.002	0.000	0.000	0.000
211	g__Niastella_s__	0.001	0.000	0.000	0.000
212	g__Nitrosomonas_s__nitrosa	0.000	0.018	0.000	0.000
213	g__Nitrosopumilus_s__	0.000	0.002	0.000	0.000
214	g__Nitrospira_s__	0.039	0.006	0.000	0.000
215	g__Novosphingobium_s__	0.066	0.007	0.166	0.019
216	g__Novosphingobium_s__capsulatum	0.006	0.000	0.000	0.000
217	g__Oceanibaculum_s__indicum	0.000	0.130	0.000	0.013
218	g__Ochrobactrum_s__	0.000	0.000	0.064	0.000
219	g__Octadecabacter_s__	0.002	0.002	0.016	0.000
220	g__Octadecabacter_s__antarcticus	0.000	0.000	0.014	0.000
221	g__Opitutus_s__	0.032	0.031	3.250	0.611
222	g__Oribacterium_s__	0.000	0.000	0.000	0.001
223	g__Paenibacillus_s__	0.000	0.000	0.000	0.002
224	g__Paludibacter_s__	0.000	0.008	0.000	0.000
225	g__Pantoea_Other	0.002	0.000	0.000	0.000
226	g__Paracoccus_s__	0.000	0.000	0.000	0.003
227	g__Paracoccus_s__marcusii	0.025	0.013	0.000	0.005
228	g__Parapedobacter_Other	0.001	0.000	0.000	0.000
229	g__Parapedobacter_s__	0.010	0.000	0.000	0.000
230	g__Parasegitibacter_s__luojiensis	0.051	0.030	0.000	0.000
231	g__Pedobacter_s__	0.038	0.001	2.249	0.043
232	g__Pedobacter_s__terricola	0.000	0.000	0.023	0.000
233	g__Pedomicrobium_s__	0.000	0.005	0.000	0.000
234	g__Pedosphaera_s__	0.001	0.000	0.000	0.000
235	g__Peptostreptococcus_s__	0.000	0.000	0.002	0.005
236	g__Peredibacter_s__starrii	0.001	0.004	0.000	0.000
237	g__Phaeobacter_Other	0.000	0.001	0.000	0.000
238	g__Phaeobacter_s__	0.000	0.002	0.001	0.002
239	g__Phaeospirillum_s__fulvum	0.000	0.003	0.000	0.000
240	g__Phenylobacterium_s__	0.002	0.017	0.060	0.014
241	g__Phormidium_s__	0.000	0.001	0.047	0.008
242	g__Phycicoccus_s__	0.002	0.000	0.000	0.000
243	g__Pigmentiphaga_s__	0.003	0.000	0.000	0.000
244	g__Pirellula_s__	0.011	0.017	0.000	0.000
245	g__Planctomyces_s__	0.122	0.010	0.000	0.012
246	g__Planctomycete_s__LF1	0.001	0.000	0.000	0.000
247	g__Planifilum_s__	0.000	0.001	0.000	0.000
248	g__Planktothrix_s__	0.000	0.007	0.000	0.000

249	g__Pleomorphomonas_Other	0.000	0.000	0.000	0.002
250	g__Pleomorphomonas_s__	0.000	0.000	0.568	0.027
251	g__Pleomorphomonas_s__oryzae	0.000	0.000	0.001	0.000
252	g__Plesiocystis_s__	0.000	0.002	0.000	0.000
253	g__Polaribacter_s__	0.000	0.000	0.010	0.000
254	g__Polaromonas_s__	0.000	0.001	0.000	0.000
255	g__Porphyromonas_s__	0.000	0.003	0.000	0.012
256	g__Prevotella_s__melaninogenica	0.000	0.000	0.000	0.017
257	g__Propionivibrio_s__	0.002	0.000	0.000	0.028
258	g__Prosthecobacter_s__	0.000	0.004	0.000	0.000
259	g__Prosthecobacter_s__debontii	0.003	0.000	0.188	0.000
260	g__PSB-M-3_s__	0.000	0.002	0.000	0.000
261	g__Pseudoalteromonas_s__	0.000	0.000	0.000	0.005
262	g__Pseudomonas_Other	0.118	0.302	0.504	1.423
263	g__Pseudomonas_s__	6.566	0.224	0.139	0.124
264	g__Pseudomonas_s__alcaligenes	0.003	0.000	0.000	0.000
265	g__Pseudomonas_s__mendocina	0.001	0.000	0.006	0.000
266	g__Pseudomonas_s__nitroreducens	0.000	0.002	0.000	0.000
267	g__Pseudomonas_s__pseudoalcaligenes	0.602	0.099	10.196	0.707
268	g__Pseudomonas_s__stutzeri	0.237	0.013	0.002	0.024
269	g__Pseudomonas_s__umsongensis	0.000	0.002	0.002	0.006
270	g__Pseudomonas_s__veronii	1.395	0.386	0.115	0.293
271	g__Pseudomonas_s__viridiflava	0.013	0.000	0.190	0.000
272	g__Pseudonocardia_s__	0.015	0.000	0.000	0.000
273	g__Pseudoxanthomonas_s__	0.000	0.000	0.061	0.000
274	g__Pseudoxanthomonas_s__mexicana	0.041	0.006	0.196	0.013
275	g__Ralstonia_s__	0.000	0.000	0.036	0.000
276	g__Rheinheimera_s__	0.213	0.129	0.719	0.065
277	g__Rhodanobacter_s__lindaniclasticus	0.000	0.003	0.000	0.000
278	g__Rhodobacter_s__	0.032	0.013	0.000	0.006
279	g__Rhodococcus_s__fascians	0.160	0.000	0.000	0.000
280	g__Rhodoferax_s__	0.001	0.008	0.116	0.843
281	g__Rhodoplanes_s__	0.210	0.117	0.009	0.044
282	g__Rhodoplanes_s__elegans	0.000	0.001	0.000	0.000
283	g__Roseivivax_s__	0.000	0.000	0.067	0.000
284	g__Roseobacter_s__denitrificans	0.000	0.000	0.040	0.000
285	g__Roseomonas_s__	0.000	0.004	0.000	0.000
286	g__Rothia_s__aeria	0.000	0.001	0.000	0.000
287	g__Rothia_s__dentocariosa	0.000	0.000	0.000	0.002
288	g__Rothia_s__mucilaginosa	0.000	0.002	0.000	0.000
289	g__Rubrivivax_s__	0.010	0.003	0.000	0.000
290	g__Sandaracinobacter_s__sibiricus	0.008	0.017	0.000	0.000
291	g__Sediminibacterium_s__	0.013	0.000	0.000	0.008
292	g__Sediminicola_s__	0.004	0.000	0.000	0.000
293	g__Serratia_s__marcescens	0.003	0.000	0.000	0.000
294	g__Shewanella_Other	0.001	0.000	0.000	0.000
295	g__Shewanella_s__	0.016	0.002	1.405	0.287
296	g__Silanimonas_s__mangrovi	0.000	0.020	0.000	0.000
297	g__Sinorhizobium_s__	0.011	0.000	0.000	0.000
298	g__Sphingobacterium_s__	0.008	0.000	0.000	0.005

299	g__Sphingobacterium_s__multivorum	0.000	0.000	0.030	0.000
300	g__Sphingobium_s__	0.000	0.017	0.415	0.017
301	g__Sphingobium_s__xenophagum	0.012	0.002	0.090	0.055
302	g__Sphingomonas_Other	0.002	0.001	0.000	0.000
303	g__Sphingomonas_s__	0.006	0.016	0.025	0.000
304	g__Sphingomonas_s__azotifigens	0.016	0.000	0.240	0.003
305	g__Sphingomonas_s__wittichii	0.000	0.000	0.000	0.010
306	g__Sphingomonas_s__yabuuchiae	0.007	0.000	0.000	0.000
307	g__Sphingopyxis_s__	0.008	0.000	0.000	0.003
308	g__Sphingopyxis_s__alaskensis	0.134	0.081	0.056	0.027
309	g__Sphingosinicella_s__microcystinivorans	0.000	0.002	0.000	0.000
310	g__Staphylococcus_Other	0.000	0.000	0.000	0.001
311	g__Staphylococcus_s__	0.030	0.057	0.081	0.138
312	g__Staphylococcus_s__epidermidis	0.000	0.000	0.001	0.000
313	g__Stenotrophomonas_s__	0.031	0.000	0.051	0.003
314	g__Steroidobacter_s__	0.009	0.019	0.000	0.000
315	g__Streptococcus_s__	0.015	0.055	0.029	0.117
316	g__Streptococcus_s__infantis	0.000	0.000	0.001	0.000
317	g__Sulfuricurvum_s__kujiense	0.042	0.084	0.000	0.000
318	g__Sulfuritalea_s__	0.021	0.001	0.000	0.000
319	g__Synechococcus_s__	0.020	0.004	0.107	0.023
320	g__Syntrophobacter_s__	0.028	0.028	0.000	0.000
321	g__Syntrophomonas_s__	0.000	0.002	0.000	0.000
322	g__Tatlockia_s__	0.003	0.000	0.000	0.000
323	g__Tepidimonas_s__	0.000	0.010	0.042	0.000
324	g__Thermomonas_s__	0.173	0.018	0.000	0.000
325	g__Thiobacillus_s__	0.206	0.369	0.000	0.000
326	g__Thiomonas_s__	0.000	0.001	0.000	0.000
327	g__Tolumonas_s__	0.005	0.002	0.000	0.000
328	g__Treponema_s__	0.000	0.001	0.000	0.000
329	g__Turneriella_s__	0.000	0.001	0.000	0.000
330	g__Ulvibacter_s__	0.000	0.000	0.040	0.000
331	g__Variovorax_s__	0.021	0.017	1.661	0.000
332	g__Veillonella_s__dispar	0.000	0.000	0.000	0.007
333	g__Vibrio_Other	0.000	0.001	0.006	0.001
334	g__Vibrio_s__	0.002	0.009	0.066	0.003
335	g__Vogesella_s__	0.004	0.000	0.000	0.000
336	g__WAL_1855D_s__	0.000	0.001	0.000	0.000
337	g__Winogradskyella_s__	0.003	0.000	0.053	0.000
338	g__Winogradskyella_s__thalassocola	0.000	0.013	0.000	0.024
339	g__Xanthobacter_s__	0.000	0.000	0.000	0.005
340	g__Yersinia_s__	0.000	0.000	0.000	0.021
341	Other_Other	0.066	0.010	0.000	0.003

Supplementary table 3. Number o bacterial-derived reads for the rhizospheres (R) and endorhizospheres (E) of the two rice varieties.

	SD20A		Pionero	
	R	E	R	E
Other_Other	33	9	215	0
g__4-29_s__	185	0	31	0
g__A17_s__	0	0	11	0
g__Abiotrophia_s__	2	0	0	0
g__Achromobacter_s__	0	31	11	274
g__Acidovorax_Other	0	0	7	11
g__Acidovorax_s__	109	0	835	538
g__Acidovorax_s__delafieldii	0	0	13	6
g__Acidovorax_s__facilis	0	0	0	2
g__Acinetobacter_s__	8	284	118	399
g__Acinetobacter_s__johnsonii	5	0	0	0
g__Acinetobacter_s__lwoffii	0	335	14	97
g__Acinetobacter_s__rhizosphaerae	0	0	49	0
g__Actinobacillus_Other	9	0	2	0
g__Actinotalea_s__	0	16	0	0
g__Adhaeribacter_s__	86	0	5	0
g__Aeromonas_Other	0	0	0	0
g__Aeromonas_s__caviae	0	6	3	36
g__Aggregatibacter_s__	6	101	0	0
g__Agrobacterium_Other	0	0	0	8
g__Agrobacterium_s__	252	1542	584	8029
g__Agrobacterium_s__undicola	0	0	0	779
g__Agrobacterium_s__vitis	0	0	0	2
g__Alcanivorax_s__	3	0	0	0
g__Algoriphagus_s__terrigena	259	723	0	0
g__Amaricoccus_s__	0	9	6	0
g__Aminobacter_s__	23	0	0	0
g__Amorphomonas_s__oryzae	2	0	0	0
g__Anaerococcus_s__	13	0	0	0
g__Anaerolinea_s__	20	0	17	0
g__Anaeromyxobacter_s__	308	0	262	0
g__Anaerovorax_s__	0	2	0	0
g__Ancylobacter_s__	0	24	108	0
g__Antarctobacter_s__	0	17	0	0
g__Aquaspirillum_s__putridiconchylium	101	0	0	0
g__Aquicella_s__	5	0	15	0
g__Aquimarina_s__	0	65	0	0
g__Aquimonas_s__	2	0	0	0
g__Arenimonas_s__	1243	0	152	0
g__Arthrobacter_Other	0	0	15	0
g__Arthrobacter_s__psychrolactophilus	0	0	96	0
g__Arthronema_s__	13	0	0	0
g__Aspromonas_s__composti	2	0	0	0
g__Asticcacaulis_Other	0	0	0	3
g__Asticcacaulis_s__	23	1559	0	1681
g__Azospira_s__	2	0	0	0
g__Azospirillum_s__	3	25	0	669
g__Azospirillum_s__massiliensis	0	390	0	1065
g__Bacillus_Other	0	0	0	59

g__Bacillus_s__	4	4	8	0
g__Bacillus_s__cereus	2	0	29	0
g__Bacteroides_s__	12	0	0	0
g__Bdellovibrio_s__	17	0	39	0
g__Bdellovibrio_s__bacteriovorus	2	0	0	0
g__Blastomonas_s__	6	0	45	0
g__Blvii28_s__	2	0	0	0
g__Bosea_s__genosp.	0	8	0	147
g__Bradyrhizobium_s__	0	0	4	4
g__Brevibacillus_s__	0	0	0	48
g__Brevibacterium_s__aureum	0	2	8	0
g__Brevundimonas_Other	1	1	0	0
g__Brevundimonas_s__diminuta	13	0	0	153
g__Bulleidia_s__moorei	0	18	0	0
g__Burkholderia_s__	0	0	0	0
g__Candidatus Endobugula_s__	0	11	0	0
g__Candidatus Koribacter_s__	71	0	247	0
g__Candidatus Nitrososphaera_s__SCA	16	0	89	0
g__Candidatus Rhabdochlamydia_s__	0	0	3	99
g__Candidatus Solibacter_s__	33	0	85	0
g__Candidatus Xiphinematobacter_s__	4	0	64	164
g__Capnocytophaga_s__	0	9	0	0
g__Capnocytophaga_s__ochracea	3	0	0	0
g__Catonella_s__	0	7	0	0
g__Caulobacter_Other	0	0	0	108
g__Caulobacter_s__	4	3013	0	3996
g__Cellulomonas_s__	0	6	0	0
g__Cellvibrio_s__	137	17167	44	48179
g__Chelativorans_s__	0	0	3	0
g__Chryseobacterium_s__	8	1	74	3568
g__Citrobacter_s__	0	235	0	0
g__Cloacibacterium_s__	100	0	246	42
g__Clostridium_Other	3	0	0	0
g__Clostridium_s__	6	0	0	0
g__Clostridium_s__acetobutylicum	5	0	0	0
g__Clostridium_s__butyricum	0	0	13	0
g__Clostridium_s__hungatei	6	0	0	0
g__Clostridium_s__intestinale	0	0	16	0
g__Coccinimonas_s__marina	0	0	47	0
g__Cohnella_s__	0	28	0	0
g__Comamonas_s__	0	0	79	0
g__Constrictibacter_s__antarcticus	7	0	0	0
g__Coprococcus_s__	0	7	0	0
g__Corynebacterium_s__	10	120	70	8
g__Corynebacterium_s__kroppenstedti	3	0	4	0
g__Crenothrix_s__	2	0	0	0
g__Crocinitomix_s__	0	0	0	170
g__Cryocola_s__	0	0	0	33
g__Cylindrospermopsis_s__	5	0	0	0
g__Cytophaga_s__	0	0	76	0

g__DA101_s__	0	0	33	0
g__DCE29_s__	1	0	10	0
g__Dechloromonas_s__	0	0	84	0
g__Defluviitalea_s__saccharophila	0	0	39	0
g__Delftia_s__	43	34	6	0
g__Demequina_s__	0	3	0	0
g__Desulfobacca_s__	56	0	0	0
g__Desulfobulbus_s__	39	0	41	0
g__Desulfococcus_s__	34	0	0	0
g__Desulfomicrobium_s__	3	0	0	0
g__Desulfomonile_s__	12	0	0	0
g__Desulforhabdus_s__amnigena	0	0	4	0
g__Desulfotalea_s__	6	0	0	0
g__Desulfovibrio_s__	20	0	8	0
g__Desulfovibrio_s__mexicanus	6	0	0	0
g__Desulfovibrio_s__putealis	7	0	33	0
g__Desulfovirga_s__adipica	20	0	14	0
g__Devosia_s__	185	382	788	4912
g__Dok59_s__	16	0	23	0
g__Dokdonella_s__	7	0	36	31
g__Dyadobacter_s__	5	0	530	40
g__Eikenella_s__	3	26	0	0
g__Endozoicomonas_s__montiporae	0	0	0	4
g__Enhydrobacter_s__	25	588	60	227
g__Enterobacter_s__	0	0	0	2
g__Epulopiscium_s__	0	0	38	0
g__Erythrobacter_Other	0	0	2	0
g__Erythrobacter_s__	0	0	191	0
g__Escherichia_s__coli	0	15	86	108
g__Euptelea_s__polyandra	0	0	0	0
g__Exiguobacterium_s__	0	0	16	102
g__Fimbriimonas_s__	9	0	33	0
g__Flavisolibacter_s__	229	0	1373	0
g__Flavobacterium_Other	0	0	8	85
g__Flavobacterium_s__	128	1909	5450	4842
g__Flavobacterium_s__frigidarium	0	3	0	0
g__Flavobacterium_s__gelidilacus	225	1191	0	318
g__Flavobacterium_s__succinicans	68	592	40	585
g__Flectobacillus_s__	4	0	0	0
g__Fluviicola_s__	39	699	70	1824
g__Francisella_s__	0	0	2	0
g__Fritschea_s__eriococci	0	0	2	0
g__Fusibacter_s__	11	0	34	0
g__Fusobacterium_s__	0	80	0	16
g__GOUTA19_s__	194	0	313	0
g__Gallionella_s__	220	0	64	0
g__Gemmatimonas_s__	6	0	0	0
g__Geobacter_s__	162	0	478	0
g__Granulicatella_s__	10	7	0	0
g__HTCC_s__	38	0	0	0

g__Haemophilus_s__parainfluenzae	51	5	7	0
g__Halomonas_s__	78	173	0	0
g__Halothiobacillus_s__	2205	0	0	0
g__Herbaspirillum_s__	0	3	0	78
g__Hydrogenophaga_s__	108	30	276	39
g__Hylemonella_s__	0	0	8	0
g__Hymenobacter_s__	0	0	8	0
g__Hyphomicrobium_Other	2	0	0	0
g__Hyphomicrobium_s__	27	0	57	0
g__Hyphomonas_s__	10	0	0	0
g__Iamia_s__	7	0	0	0
g__Janthinobacterium_s__	0	17	24	10
g__Janthinobacterium_s__lividum	0	0	78	98
g__K82_s__	4	0	0	0
g__Kaistia_s__	8	6	0	0
g__Kaistobacter_s__	381	0	1067	0
g__Klebsiella_s__	5	0	0	68
g__Kocuria_s__rhizophila	0	8	0	0
g__LCP-6_s__	116	0	50	0
g__Lacibacter_s__cauensis	134	59	341	0
g__Lactobacillus_s__zeae	0	0	0	18
g__Leadbetterella_s__	90	0	60	0
g__Leptolyngbya_s__	10	0	0	0
g__Leptonema_s__	6	0	0	0
g__Leptospira_s__	9	0	13	0
g__Leptotrichia_s__	0	0	0	34
g__Leuconostoc_s__	0	2	0	0
g__Limnobacter_s__	1042	0	18419	858
g__Limnohabitans_s__	3	0	2	18
g__Loktanella_s__	0	7	0	34
g__Luteimonas_s__	11	0	40	0
g__Luteolibacter_s__	5	4	21	550
g__Lutibacterium_s__	0	0	0	787
g__Lutimonas_s__	3	23	0	0
g__Lysobacter_s__	16	36	10	191
g__Magnetospirillum_s__	0	10	0	0
g__Maribacter_s__	0	0	2	0
g__Marinobacter_s__	0	0	703	0
g__Marinobacter_s__bryozoorum	0	0	4	0
g__Massilia_s__haematophila	0	0	2	0
g__Mesorhizobium_s__	13	0	0	0
g__Methylibium_s__	17	0	20	0
g__Methylobacterium_s__	4	0	20	0
g__Methylomicrobium_s__	8	0	0	0
g__Methylomicrobium_s__agile	37	0	0	0
g__Methylophaga_s__	1668	0	0	0
g__Methylotenera_s__mobilis	91	0	9376	490
g__Methyloversatilis_s__	174	21	127	6
g__Methylovorus_s__glucosotrophus	0	0	0	154
g__Micrococcus_s__	0	2	0	0

g__Microvirgula_s__aerodenitrificans	2	0	0	8210
g__Muricola_s__jejuensis	0	0	0	44
g__Mycoplana_s__	787	748	741	2903
g__Mycoplasma_s__	3	0	0	0
g__Myxococcus_s__	20	0	0	0
g__Nautella_s__	0	0	0	16
g__Neisseria_s__	12	37	54	0
g__Neisseria_s__oralis	3	0	0	0
g__Neisseria_s__subflava	50	24	0	0
g__Nevskia_s__ramosa	38	85	224	852
g__Niabella_s__	0	0	8	0
g__Niastella_s__	0	0	2	0
g__Nitrosomonas_s__nitrosa	59	0	0	0
g__Nitrosopumilus_s__	5	0	0	0
g__Nitrospira_s__	19	0	126	0
g__Novosphingobium_s__	23	63	215	543
g__Novosphingobium_s__capsulatum	0	0	19	0
g__Oceanibaculum_s__indicum	425	42	0	0
g__Ochrobactrum_s__	0	0	0	210
g__Octadecabacter_s__	5	0	5	52
g__Octadecabacter_s__antarcticus	0	0	0	46
g__Opitutus_s__	102	1997	105	10611
g__Oribacterium_s__	0	4	0	0
g__PSB-M-3_s__	7	0	0	0
g__Paenibacillus_s__	0	7	0	0
g__Paludibacter_s__	27	0	0	0
g__Pantoea_Other	0	0	7	0
g__Paracoccus_s__	0	9	0	0
g__Paracoccus_s__marcusii	41	16	80	0
g__Parapedobacter_Other	0	0	2	0
g__Parapedobacter_s__	0	0	34	0
g__Parasegitibacter_s__luojiensis	98	0	165	0
g__Pedobacter_s__	3	140	123	7345
g__Pedobacter_s__terricola	0	0	0	74
g__Pedomicrobium_s__	15	0	0	0
g__Pedosphaera_s__	0	0	2	0
g__Peptostreptococcus_s__	0	16	0	7
g__Peredibacter_s__starrii	12	0	2	0
g__Phaeobacter_Other	2	0	0	0
g__Phaeobacter_s__	7	5	0	4
g__Phaeospirillum_s__fulvum	9	0	0	0
g__Phenylobacterium_s__	54	45	6	196
g__Phormidium_s__	3	27	0	153
g__Phycicoccus_s__	0	0	6	0
g__Pigmentiphaga_s__	0	0	11	0
g__Pirellula_s__	57	0	37	0
g__Planctomyces_s__	33	38	397	0
g__Planctomycete_s__LF1	0	0	3	0
g__Planifilum_s__	4	0	0	0
g__Planktothrix_s__	23	0	0	0

g__Pleomorphomonas_Other	0	5	0	0
g__Pleomorphomonas_s__	0	87	0	1855
g__Pleomorphomonas_s__oryzae	0	0	0	4
g__Plesiocystis_s__	5	0	0	0
g__Polaribacter_s__	0	0	0	32
g__Polaromonas_s__	4	0	0	0
g__Porphyromonas_s__	11	39	0	0
g__Prevotella_s__melaninogenica	0	55	0	0
g__Propionivibrio_s__	0	93	5	0
g__Prosthecobacter_s__	12	0	0	0
g__Prosthecobacter_s__debontii	0	0	9	615
g__Pseudoalteromonas_s__	0	15	0	0
g__Pseudomonas_Other	987	4646	385	1646
g__Pseudomonas_s__	733	406	21438	453
g__Pseudomonas_s__alcaligenes	0	0	9	0
g__Pseudomonas_s__mendocina	0	0	2	21
g__Pseudomonas_s__nitroreducens	7	0	0	0
g__Pseudomonas_s__pseudoalcaligen	322	2310	1965	33292
g__Pseudomonas_s__stutzeri	42	78	773	5
g__Pseudomonas_s__umsongensis	5	18	0	6
g__Pseudomonas_s__veronii	1262	957	4554	374
g__Pseudomonas_s__viridiflava	0	0	43	619
g__Pseudonocardia_s__	0	0	48	0
g__Pseudoxanthomonas_s__	0	0	0	200
g__Pseudoxanthomonas_s__mexicana	19	44	133	641
g__Ralstonia_s__	0	0	0	119
g__Rheinheimera_s__	421	212	696	2349
g__Rhodanobacter_s__lindaniclasticus	10	0	0	0
g__Rhodobacter_s__	42	18	104	0
g__Rhodococcus_s__fascians	0	0	524	0
g__Rhodoferax_s__	26	2754	3	379
g__Rhodoplanes_s__	381	143	686	31
g__Rhodoplanes_s__elegans	3	0	0	0
g__Roseivivax_s__	0	0	0	220
g__Roseobacter_s__denitrificans	0	0	0	129
g__Roseomonas_s__	13	0	0	0
g__Rothia_s__aeria	2	0	0	0
g__Rothia_s__dentocariosa	0	7	0	0
g__Rothia_s__mucilaginosa	7	0	0	0
g__Rubrivivax_s__	9	0	34	0
g__Sandaracinobacter_s__sibiricus	54	0	27	0
g__Sediminibacterium_s__	0	26	42	0
g__Sediminicola_s__	0	0	12	0
g__Serratia_s__marcescens	0	0	9	0
g__Shewanella_Other	0	0	3	0
g__Shewanella_s__	8	938	53	4588
g__Silanimonas_s__mangrovi	66	0	0	0
g__Sinorhizobium_s__	0	0	37	0
g__Sphingobacterium_s__	0	17	26	0
g__Sphingobacterium_s__multivorum	0	0	0	99

g__Sphingobium_s__	54	56	0	1356
g__Sphingobium_s__xenophagum	7	180	38	294
g__Sphingomonas_Other	3	0	8	0
g__Sphingomonas_s__	51	0	20	81
g__Sphingomonas_s__azotifigens	0	10	53	784
g__Sphingomonas_s__wittichii	0	34	0	0
g__Sphingomonas_s__yabuuchiae	0	0	22	0
g__Sphingopyxis_s__	0	10	26	0
g__Sphingopyxis_s__alaskensis	264	88	439	184
g__Sphingosinicella_s__microcystinivo	6	0	0	0
g__Staphylococcus_Other	0	4	0	0
g__Staphylococcus_s__	185	450	97	266
g__Staphylococcus_s__epidermidis	0	0	0	3
g__Stenotrophomonas_s__	0	10	102	167
g__Steroidobacter_s__	62	0	30	0
g__Streptococcus_s__	180	382	48	94
g__Streptococcus_s__infantis	0	0	0	2
g__Sulfuricurvum_s__kujiense	275	0	138	0
g__Sulfuritalea_s__	3	0	67	0
g__Synechococcus_s__	13	74	65	349
g__Syntrophobacter_s__	93	0	92	0
g__Syntrophomonas_s__	5	0	0	0
g__Tatlockia_s__	0	0	10	0
g__Tepidimonas_s__	32	0	0	138
g__Thermomonas_s__	58	0	566	0
g__Thiobacillus_s__	1206	0	673	0
g__Thiomonas_s__	3	0	0	0
g__Tolumonas_s__	6	0	17	0
g__Treponema_s__	4	0	0	0
g__Turneriella_s__	2	0	0	0
g__Ulvibacter_s__	0	0	0	131
g__Variovorax_s__	57	0	69	5424
g__Veillonella_s__dispar	0	23	0	0
g__Vibrio_Other	3	3	0	18
g__Vibrio_s__	31	10	6	217
g__Vogesella_s__	0	0	14	0
g__WAL_1855D_s__	3	0	0	0
g__Winogradskyella_s__	0	0	11	173
g__Winogradskyella_s__thalassocola	44	79	0	0
g__Xanthobacter_s__	0	15	0	0
g__Yersinia_s__	0	67	0	0
g___s__	0	0	60	0

References

71. Caporaso, J.G.; Lauber, C.L.; Walters, W.A.; Berg-Lyons, D.; Lozupone, C.A.; Turnbaugh, P.J.; Fierer, N.; Knight, R. Global patterns of 16S rRNA diversity at a depth of millions of sequences per sample. *Proc. Natl. Acad. Sci. USA* **2011**, *108*, 4516–4522.
72. Claesson, M.J.; O'Sullivan, O.; Wang, Q.; Nillila, J.; Marchesi, J.R.; Smidt, H.; de Vos, W.M.; Ross, R.P.; O'Toole, P.W. Comparative analysis of pyrosequencing and a phylogenetic microarray for exploring microbial community structures in the human distal intestine. *PLoS ONE* **2009**, *4*, e6669.
